# Discovery and characterization of a novel telomerase alternative splicing isoform that protects lung cancer cells from chemotherapy induced cell death

**DOI:** 10.1038/s41598-025-90639-3

**Published:** 2025-02-25

**Authors:** Jeongjin J. Kim, Alexander Ahn, Jeffrey Y. Ying, Andrew T. Ludlow

**Affiliations:** School of Kinesiology, University of Michigan, Ann Arbor, MI 48109 USA

**Keywords:** Alternative RNA splicing, Telomerase, TERT, Lung cancer, Cancer, Cell biology

## Abstract

All cancer cells must adopt a telomere maintenance mechanism to achieve replicative immortality. Most human cancer cells utilize the enzyme telomerase to maintain telomeres. Alternative splicing of TERT regulates the amount and function of telomerase, however many alternative splicing isoforms of TERT have unknown functions. Single molecule long read RNA/cDNA sequencing of TERT revealed 45 *TERT* mRNA variants including 13 known and 32 novel variants. Among the variants, *TERT* Delta 2–4, which lacks exons 2–4 but retains the original open reading frame, was selected for further study. Induced pluripotent stem cells and cancer cells express higher levels of *TERT* Delta 2–4 compared to primary human bronchial epithelial cells. Overexpression of TERT Delta 2–4 enhanced clonogenicity and resistance to cisplatin-induced cell death. Knockdown of endogenous TERT Delta 2–4 in Calu-6 cells reduced clonogenicity and resistance to cisplatin. Our results suggest that TERT Delta 2–4 enhances cancer cells’ resistance to cell death. RNA sequencing following knockdown of Delta 2–4 TERT indicates that translation is downregulated and that mitochondrial related proteins are upregulated compared to controls. Overall, our data indicate that TERT produces many isoforms that influence the function of TERT and the abundance and activity of telomerase.

## Introduction

**Fig. 1:**
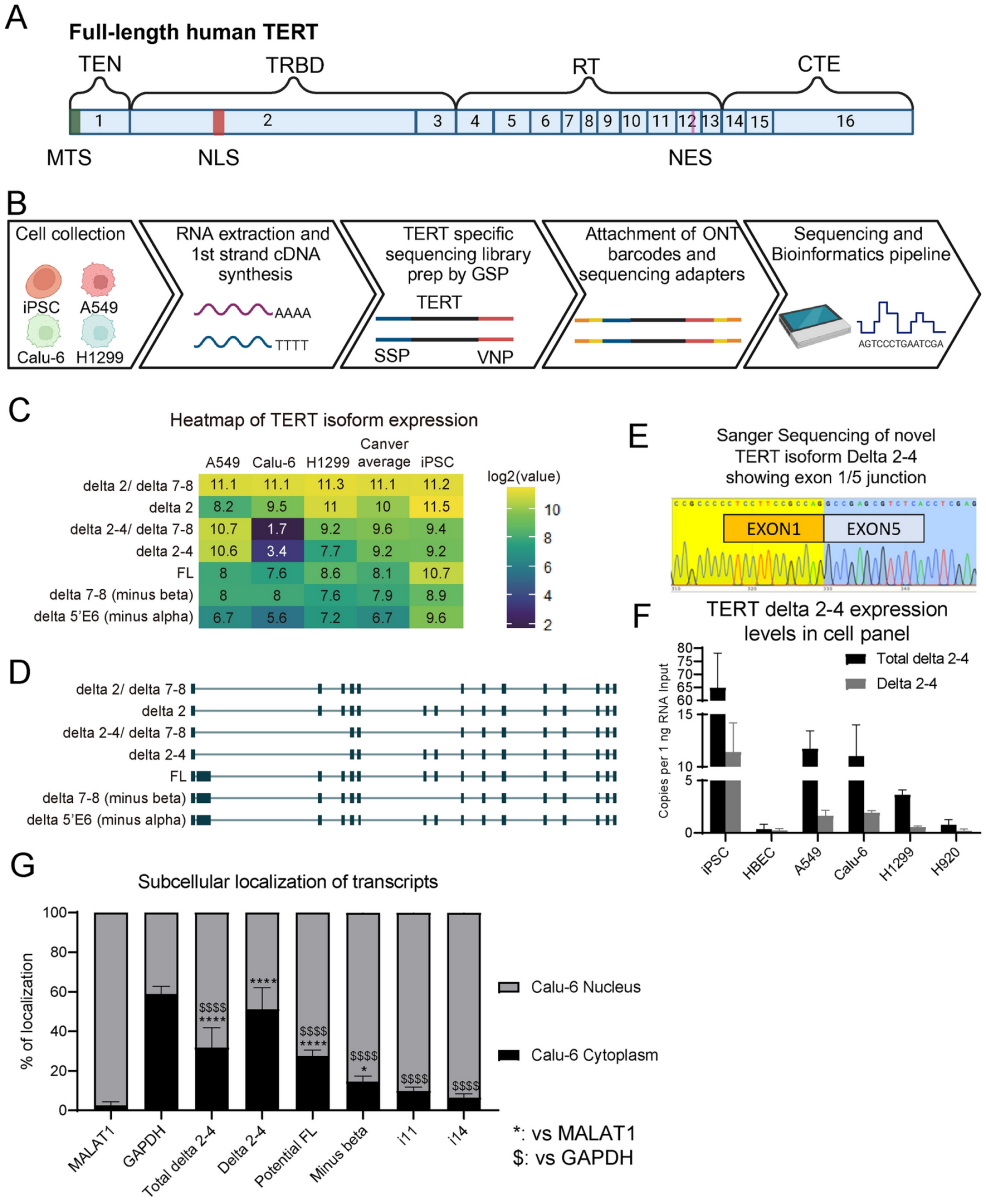
Long-read sequencing pipeline to discover novel TERT Delta 2–4 isoform and validation of Delta 2–4 expression. (**A**) Cartoon of full-length TERT exons. Four functional domains (*TEN* telomerase N-terminal, *TRBD* telomerase RNA-binding domain, *RT* reverse transcriptase, *CTE* C-terminal extension) and three localization signals (*MTS* mitochondrial-targeting signal in exon 1, *NLS* nuclear localization signal in exon2, *NES* nuclear export signal in exon 12) are shown. (**B**) Long-read sequencing pipeline to discover novel TERT isoforms. Four cell lines: induced pluripotent stem cells (iPSC-CHiPSC22), Calu-6, A549, and H1299 were pelleted from three biological replicates and RNA was extracted from each pellet. First strand cDNA was synthesized with oligo dT using superscript 4, followed by PCR with gene specific primers (GSP; TERT exons targeting primers) tailed with SSP or VNP (SSP-Exon 1 or VNP-Exon 16). After size exclusion of the TERT specific library, ONT (Oxford nanopore technology) barcodes and rapid 1D sequencing adapters were attached. Samples were loaded into MinION Mk1C and raw sequence files were processed by a bioinformatics pipeline consisting of Guppy, minimap2, TranscriptClean, TALON, SWAN, and R. (**C**) Heatmap showing expression levels of relatively abundant TERT isoforms. Numeric values are Log2-transformed TPM values and colors indicate expression level. Cancer average is the average of A549, Calu-6, and H1299. (**D**) Transcript models of the isoform (Left to right: 5ʹ to 3ʹ). Boxes indicate exons and solid lines between exons indicate introns. (**E**) Sanger Sequencing result confirmed TERT exon 2–4 splicing event from full length cDNA clone in pTOPO vector. (**F**) TERT Delta 2–4 expression levels in cell panels (determined by ddPCR; n = 3 biological replicates per condition). Total delta 2–4 indicates both exons 2–4 skipping variants including Delta 2–4 and delta2-4/delta 7–8, and Delta 2–4 indicates exons 2–4 skipping with exon 7/8 inclusion (note the capital D) (determined by ddPCR; n = 3 biological replicates per condition). (**G**) Percentage of localization between cytoplasm and nucleus was determined for nuclear non-coding RNA MALAT1, GAPDH, and TERT isoforms. Total delta 2–4: Delta 2–4 and delta 2–4/delta 7–8; Delta 2–4: exons 2–4 skipping with exons 7/8; Potential FL: exons 7/8 including TERT; Minus beta: exons 6/9 junction containing TERT; i11: intron 11 detained TERT; i14: intron 14 detained TERT (TERT isoforms: determined by ddPCR; MALAT1 and GAPDH: determined by gel-based PCR; n = 3 biological replicates). Unpaired t-test was performed to compare TERT mRNA variants’ percentage of localization compared to MALAT1 or GAPDH (^$$$$^*p* < 0.0001 compared to MALAT1; ****p < 0.0001 and *p < 0.05 compared to GAPDH). Data are presented as means ± standard deviations where applicable.

Telomerase is a ribonucleoprotein enzyme composed of a protein subunit TERT (Telomerase reverse transcriptase), RNA template TERC (Telomerase RNA component; also called TR), and accessary proteins^1^. The primary function of telomerase is to maintain or elongate telomeres by de novo telomere synthesis^2^. TERT has four protein domains (N-terminal extension, TEN; Telomerase RNA binding domain, TRBD; reverse transcriptase domain, RT; C-terminal extension, CTE). All four protein domains are required for telomerase to be able to synthesize telomere repeats^3,4^. Three localization signals are embedded in TERT’s exons including a mitochondrial targeting signal (MTS) in exon 1^5^, a nuclear localization signal (NLS) in exon 2^6^, and a nuclear export signal (NES) in exon 12^7^ (Fig. 1A). These signals play important roles in dictating the function of TERT proteins in different cell types and under different physiological conditions.

*TERT* is a 16-exon gene that when spliced to full-length *TERT* (FL *TERT*) generates active telomerase^8^. However, *TERT* pre-mRNAs undergo alternative splicing (AS) and can form *TERT* mRNA variants in addition to FL *TERT*^9^. There are over 20 alternative splicing variants of TERT that have been previously studied^10–17^. TERT alternative splicing not only produces *TERT* mRNA variants and protein isoforms, but it also regulates the subcellular localization of *TERT* pre-mRNAs and mRNAs^18^. Of the *TERT* variants that have been studied, none have been found to have telomerase activity. Minus beta (lacking exons 7–8), minus alpha (cryptic 5ʹ splice site in exon 6 removing 36 nucleotides), minus gamma (lacking exon 11), INS3 (modifications in intron 14-exon 16), and INS4 (modifications of intron 14–exon 16) have all been identified as likely lowly expressed dominant-negative isoforms^10,13–15^. However, the functions of many TERT isoforms remain unknown. The major technical roadblock to studying AS regulation of TERT is the lack of single molecule techniques for detecting multiple splicing events on individual mRNA molecules. For example, although single exon (e.g., exon 2, exons 7–8, and exon 11) splicing events are readily detected by RT-PCR, whether multiple splicing events occur on the same mRNA or on independent mRNAs is unknown. In fact, technological advancements have increased the throughput of full-length single molecule sequencing allowing for multiple cell lines to be profiled at once compared to the single cell lines that have been used in the past to study *TERT* alternative splicing variants^16,17^. By deciphering the catalogue of *TERT* mRNA variants across various cell types and determining the functions of TERT protein isoforms, we may be able to determine a means to target TERT specifically in cancer cells while sparing TERT functions in normal adult stem cells and other TERT positive cell types.

To begin to catalogue *TERT* mRNA variants, we generated *TERT* specific sequencing libraries from four telomerase positive human cell lines and sequenced them on an Oxford Nanopore Technologies (ONT) single molecule long-read sequencer. As a result, we detected 45 variants including 13 known and 32 novel *TERT* mRNA variants. Among the variants, we chose a newly identified *TERT* mRNA lacking exons 2–4 (we call TERT Delta 2–4) that maintained the original open reading frame of TERT. We determined that it was expressed in lung cancer cells and human induced pluripotent stem cells but not in normal human bronchial epithelial cells. Also, we identified that TERT Delta 2–4 promotes colony formation and protects lung cancer cells from chemotherapy (cisplatin).

## Materials and methods

### MinION TERT sequencing library preparation and sequencing

RNA was extracted (Qiazol) from four human cell lines (CHiPSC22 Takara, H1299, Calu-6, A549). cDNAs were synthesized (SuperScript IV Thermo Fisher). Sequencing libraries were prepared following ONT kit instructions with TERT specific primers with ONT sequences added on for a total of 30 cycles. DNA bands in the 2.5–4 kb range (upper bands; upper TERT) and bands below 2.5 to 0.5 kb (lower bands; lower TERT) were excised and purified via gel extraction (Qiagen). Next, the two size binned fractions were amplified with ONT barcoding primers 1–12 (4 cell lines by 3 replicates for each size bin) for an additional 20 cycles. Barcoded libraries from each cell line were pooled at equal molar ratios for each size bin (upper bands or lower bands). The pooled libraries for each size bin were sequenced on separate flow cells (one for upper bands and one for lower bands; R9.4.1 flowcells). Sequencing was performed for the standard 72 h sequencing protocol run on the MinION Mk1C, using the MinKNOW software (v 3.3.2). From the lower TERT libraries, 21.8 million reads were obtained and 16.4 million reads passed from all 12 samples. From the upper TERT libraries, 14.1 million reads were obtained, and 12.2 million reads passed from all 12 samples. Base-calling was performed by Guppy (v 3.1.5). Fastq files which were classified as passed (Phred score of 8) by the MinKNOW software were subsequently processed and analyzed.

### Bioinformatics analyses of TERT sequencing libraries

Passed fastq files were aligned to the human genome (GRCh38.p13) using minimap2 (v 2.14^19,20^) in SAM MD-tag aware mode. Sam files were sorted with SAMtools (v 1.13^21^) followed by transcriptClean (v 2.0.2^22^) for correction of read microindels (< 5 bp), mismatches, and noncanonical splice junctions (< 5 bp). Read annotation was performed with TALON (v 5.0^23^), using the human Gencode v.38 reference annotation gtf file with minimum alignment identity = 0.5 and coverage = 0.5. Identified transcripts were subsequently filtered using a minimum count threshold of N = 35 reads in K = 2 samples. Based on the quantification, Swan (v 2.0^24^) was used to further process the data. Analyses of the data were performed using python (v 3.9) and R studio (v 2022.07.01^25^) with the reshape2 package (v1.4.4^26^), dplyr package (v 1.0.6^27^), and ggplot2 package (v 3.3.6^28^).

### Bioinformatics analyses of Delta 2–4 knockdown and rescue

Calu-6 cell lines with stable expression of either empty Flag-V5 or Flag-V5-Delta 2–4 were treated with scramble non-targeting siRNA controls or an siRNA targeting Delta 2–4 TERT for 48 h. Long-read sequencing (cDNA) libraries were prepared, and sequencing was performed on a MinION Mk1c (see Supplemental Methods for details). Fastq files were aligned to the human genome (GRCh38.p14) and processed as described above using minimap2 (v 2.14^19,20^), SAMtools (v 1.13^21^), and transcriptClean (v 2.0.3^22^). Read annotation was performed with TALON (v 5.0^23^), using the human Gencode v.44 reference annotation gtf file with minimum alignment identity = 0.6 and coverage = 0.8. Identified transcripts were subsequently filtered using a minimum count threshold of N = 5 reads in K = 3 samples. The filtered count matrix generated by TALON (details are described in supplementary material) was loaded into DESeq2 for the further analysis (v.1.40.2^29^). To generate a sample distance heatmap (Euclidean distance), pcaExplorer (v.2.26.1^30,31^) was used. Differentially expressed genes (DEGs) were identified and vsd transformed values of the genes were used to generate a gene expression heatmap using pheatmap (v.1.0.12^32^) and viridis (v.0.6.5;^33^). To generate a volcanoplot, p-values and log-fold change (LFC) values from differential analysis was used with EnhancedVolcano (v.1.18.0^34^), with cut-off being LFC > 1.5 and *p* value < 0.01. Gene set enrichment analysis was carried out with DAVID^35,36^ using a list of differentially expressed genes. Analyses of the data were performed using R studio (v 2023.06.2^25^).

### Cell culture and cell lines

A Cellartis® Human iPSC Line from Takara (ChiPSC22, Cat. No. Y00320) was cultured with strict adherence to manufacturer’s protocols and manuals. Cellartis® DEF-CS 500 (Y30017) culture system was employed to maintain iPSC cultures (thawing, passages, media changes and cryopreservation). NSCLC cell lines (A549 (RRID:CVCL_0023), Calu-6 (RRID:CVCL_0236), NCI-H1299 (RRID:CVCL_0060), and NCI-H920 (RRID:CVCL_1599)) and Human Bone Osteosarcoma Epithelial Cells (U-2 OS (RRID:CVCL_0042)) were maintained in culture at 37 °C in 5% CO_2_ in 4:1 DMEM:Medium 199 supplemented with 10% cosmic calf serum (HyClone, Logan, UT). All unmodified cell lines were obtained from American Type Culture Collection (ATCC, Manassas, VA). Human bronchial epithelial cells (HBEC, primary ATCC—PCS-300-010) were maintained in bronchial epithelial growth media (ATCC—PCS-300-030) supplemented with a bronchial epithelial cell growth kit (ATCC—PCS-300-040) on collagen coated plates (porcine gelatin, Millipore Sigma). Cell line identity was verified by the vendor (ATCC). All cell lines were confirmed to be myocoplasma free at the start of the culture and several clean vials were frozen back for subsequent use (e-Myco kit, Bulldog-Bio). Cells were continuously cultured for 70 passages or up to three months, whichever occurred first, at which point a new mycoplasma free vial was obtained, thawed and cultured.

### Plasmids

A codon-optimized Delta 2–4 construct was generated (GeneArt Gene Synthesis, Invitrogen) based on the TERT mRNA sequence (NM_198253.3). The Delta 2–4 sequence was initially inserted into pDONR221, then cloned into pLenti6.2-3xFLAG-V5-ccdB plasmid by Gateway cloning LR recombination reaction (Cat no. 11791019, Invitrogen™) to produce 3xFLAG-V5-Delta 2–4 plasmid. pLenti6.2-3xFLAG-V5-ccdB was a gift from Susan Lindquist & Mikko Taipale (Addgene plasmid # 87072; http://n2t.net/addgene:87072; RRID:Addgene_87072^37^).

### Stable cell line generation

Stable cell lines expressing Flag-V5-Delta 2–4 were generated for Calu-6 and U-2 OS cells. Lentivirus was produced by transfecting 293T cells (RRID:CVCL_0063) with either pLenti6.2-3xFLAG-V5-ccdB (Control empty vector) or 3xFLAG-V5-Delta 2–4 plasmid, and helper plasmids (psPAX, pMD). Viral supernatants were collected, and target cells (Calu-6 and U-2 OS) were infected. Following infection cells were selected with Blasticidin (Cat no. R210-01, Gibco) and populations of stably selected cells were cultured and utilized in subsequent experiments.

### Transient siRNA experiments

Calu-6 cells were plated in 6-well plates (300,000 cells per well) and were transfected with non-silencing controls (Santa Cruz Biotechnology, sc-37007) or siRNAs targeting TERT exons 1/5 junction (IDT Integrated DNA Technologies; siD2-4 (12:12), siD2-4 (8:16), siD2-4 (16:8) in Table S3). Calu-6 cells were transfected with 10 nM of siRNA using Opti-MEM (Gibco) and RNAi max (Invitrogen) for either 48-or-72 h. Following transfections, cells were washed, trypsinized, counted, and pelleted for downstream analysis.

### Reverse transcription-ddPCR

RNA was extracted from frozen cell pellets using RNeasy® Plus Universal Mini Kit (Qiagen, 73404) according to manufacturer’s protocol. 1 µg of RNA was used to synthesize cDNAs (SuperScript IV, Thermo Fisher). All cDNAs were diluted 1:4 (20 µL of cDNA + 60 µL nuclease-free water) before use. The cDNAs were used within 48 h of production in ddPCR measures and stored at − 20 °C thereafter. Detailed information about PCR conditions, primers, probes, and reagents are included in the “Detailed methods” section of the supplementary material and our previous publication^38^.

### Cloning of TERT mRNA variants

Using oligo dT primed cDNAs from A549 cells (total RNA input 2 µg, Superscript IV), PCR was performed with primers targeting TERT exon 1 and 16 (TERT Ex1 Forward and TERT Ex16 Reverse; Sequences are in Table S1) and a high-yield and high-fidelity polymerase (Advantage® GC 2 PCR Kit, Cat. No. 639120, TaKaRa). The PCR products were TA cloned into TOPO™ TA and transformation was performed using TOP 10 E.coli (Termo Fisher). EcoRI digests identified clones that had inserts shorter than FL TERT. Identified unique plasmids were Sanger sequenced with M13 F and M13 R to verify sequences and aligned to TERT using freely available sequence analysis tools. Delta 2–4 and Delta 2–4/delta 7–8 TERT were identified using these methods.

### Nuclear and cytoplasm fragmentation

Calu-6 cells (n = 3) were used to obtain cytoplasmic RNA and nuclear RNA fractions using PARIS™ Kit (Cat no. AM1921, Invitrogen™). Following RNA extraction, TURBO DNA-free™ Kit (Cat no. AM1907, Invitrogen™) was used to remove trace DNA contamination. Cytoplasmic and nuclear localization of MALAT1 and GAPDH were measured by gel-based PCR using 2 µL of cDNA (equivalent to 25 ng of RNA input) and 2× EmeraldAmp® MAX HS PCR Master Mix (Cat no. RR330, TaKaRa). Localization of TERT transcripts was measured by ddPCR as described above. Primer sequences to target MALAT1 and GAPDH can be found in Table S1.

### Telomerase activity determined by droplet digital TRAP assay

Quantification of telomerase enzyme activity was determined by the droplet digital TRAP assay^39,40^. In brief, a 1.0 × 10^6^ cell pellet was lysed in 40 µL of NP-40 lysis buffer, diluted to a final concentration of 1.25 × 10^3^ cells/µL, and 2 µL added to an extension reaction (50 cell equivalents per µL) for 60 min followed by a 5 min heat inactivation of telomerase at 95 °C. An aliquot (2 µL) of extension products containing an equivalent of 100 cells was amplified in a droplet digital PCR for 40 cycles. Droplet fluorescence intensity and number were read and counted on the QX200 Droplet Reader (Bio-Rad). Data were calculated to represent telomerase extension products per cell equivalent.

### Telomere length analysis by terminal restriction fragment assay

The average length of telomeres (terminal restriction fragment lengths) was measured as previously described^41^ with the following modifications: A DIG-labeled DNA molecular weight marker II ladder was loaded on either side of the samples (Millipore Sigma, St. Louis, MO). DNA was transferred to Hybond-N + membranes (GE Healthcare, Piscataway, NJ) using overnight transfers. The membrane was briefly air-dried and DNA was fixed by UV-crosslinking. Membranes were then probed for telomeres using a digoxigenin (DIG)-labeled telomere probe, detected with a horseradish peroxidase-linked anti-DIG antibody (Cat no. 11093274910; Roche, Basel, Switzerland, RRID:AB_2734716), exposed with CDP-star (Cat no. 11759051001; Roche), and imaged (Chemidoc XRS + Molecular Imager, Bio-Rad).

### Clonogenic assay

Calu-6 and U-2 OS cells with or without TERT Delta 2–4 overexpression were used for clonogenic assays. Calu-6 cells were treated with siRNAs (control and Delta 2–4 targeting) in duplicate (10 nM siRNA). After 24 h of siRNA and treatments, cells were trypsinized, counted, and plated in triplicate at densities of 100 cells per well in a 6-well plate resulting in 6 replicates for each condition. Media was changed every four days, and once visible colonies were present (~ 11 days), they were fixed and stained with Crystal violet^38^. Plates were imaged (Chemidoc XRS + Molecular Imager, Bio-Rad) and the number of colonies were counted by ColonyCountJ^42^.

### Alamar blue cell viability assay

To determine the viability of cells with overexpression of Delta 2–4, RNAi and cisplatin treatment, we used Alamar blue assays. In TERT Delta 2–4 expressing U-2 OS and Calu-6 cells, 1.0 × 10^4^ cells were plated on 96-well plates in 180 µL of growth media. Immediately after plating the cells, cells were treated with control (PBS, vehicle) or cisplatin (Cat. No. 232120-50MG, Millipore Sigma) dissolved in PBS for 48 h. Following the 48 h of cisplatin or vehicle treatment cells were incubated with Almar blue, after 4 h of incubation with Alamar blue, the viability (Alamar blue, Cat. No. DAL1025, Invitrogen™) of each well was determined on a plate reader (Molecular Devices SpectraMax iD3).

### Western blot analysis

Cell pellets (1 × 10^6^ cells) were collected and lysed in 100 µL of NP40 lysis buffer. Total protein lysates were further treated with 100 µL of 2 × Laemmli buffer (Bio-Rad) and boiled for 10 min at 95 °C. Each protein lysate was loaded by equal volume and resolved by SDS–polyacrylamide gel electrophoresis, transferred to polyvinylidene fluoride (PVDF) membranes, and detected with antibodies for TERT (rabbit monoclonal, Y182, Cat. No. ab32020, Abcam, 1:1 000 dilution in 5% BSA), FLAG (rabbit monoclonal, D6W5B, Cat. No. 14973, Cell Signaling, 1:1 000 dilution in 5% BSA), and V5 (mouse monoclonal, SV5-Pk1, Cat. No. R960-25, 1:1 000 dilution in 5% BSA). Protein loading was determined with antibodies against beta-actin (mouse monoclonal [8H10D10], 3700, Cell Signaling Technology, 1:1 000 in 5% BSA). Blots were imaged with Bio-Rad ChemiDoc XRS + Molecular Imager and quantified with Bio-Rad Image Lab software.

### Statistical analysis

For *TERT* transcript subcellular localization experiment (Fig. 1G) and siRNA testing experiments (Fig. S2B–D), one-way ANOVA with uncorrected Fisher’s LSD for post hoc comparisons were used to determine statistical significance between experimental groups. For knockdown and rescue experiments (Figs. 3D,E, 4D, Fig. S3D,E), p-value was calculated by either Tukey’s or Šídák’s multiple comparisons test following one-way ANOVA. When a *p*-value from a comparison between two groups was *p* < 0.05, the two groups were assigned different letters of the alphabet (i.e., a vs. b). When the *p*-value was *p* ≥ 0.05, two groups were assigned to the same letter (a vs. a). To compare two conditions, unpaired two-tailed t-test was used to calculate *p*-values.

## Results

### Long-read sequencing identifies the catalogue of TERT mRNA variants

To capture the *TERT* mRNA variant expression profile, a TERT specific sequencing library was prepared with four different cell lines: human induced pluripotent stem cells (iPSC), and three non-small cell lung cancer (NSCLC) cell lines (A549, Calu-6, and H1299; Fig. 1B). We observed 45 *TERT* mRNA variants and generated a heatmap to show log2-transformed expression levels in the four cell lines (Fig. S1A). In addition to the heatmap, characteristics of the TERT isoforms were determined such as the open reading frame (ORF), premature termination codon (PTC), novelty, and a transcript model (exons and introns) (Fig. S1B). Among the discovered *TERT* mRNA variants, we focused on more abundant variants that are located near the top of the heatmap. Most of the abundant *TERT* variants were known mRNAs, such as delta 2 TERT (also known as Del2 which lacks exon 2), delta 2 excluding exons 7–8 (delta 2/delta 7–8), FL TERT (full-length with all 16 exons), delta 7–8 TERT (known as minus beta, skipping of exons 7–8), and delta 5’E6 TERT (known as minus alpha, which uses a cryptic splice site in exon 6 that removes that first 36 nucleotides of exon 6; Fig. 1C,D). However, we also discovered a relatively abundant novel *TERT* splicing event that was present in all cell lines: the skipping of exons 2–4 (Fig. 1C,D). We observed two abundant delta 2–4 mRNA variants: one variant skipped exons 2–4 and included exons 7–8 and retained the original open reading frame (referred to herein as Delta 2–4, note the capital “D”). The second variant skipped exons 2–4 and was combined with the beta deletion (skipping of exons 7–8; referred to herein as delta 2–4/delta 7–8 in Fig. 1C,D). We also identified these Delta 2–4 variants with an independent approach based on traditional cDNA cloning. Delta 2–4 was identified in several clones by restriction enzyme digests and validated by Sanger sequencing, supporting our findings from the long-read RNA sequencing (Fig. 1E). To quantitatively measure the expression level of exons 2–4 skipping mRNA variants, we developed two droplet digital PCR (ddPCR) assays. One assay (referred to as “total delta 2–4”) detects both Delta 2–4 (with exons 7/8) and delta 2–4/delta 7–8 (without exons 7/8) by using primers in TERT exon 1 and exon 5. The second assay detects only Delta 2–4 including exons 7 and 8 by using primers in TERT exon 1 and exon 7 (Fig. S1C). A probe was designed to detect the exon 1/5 junction and was used for both assays to make the assays specific to the exons 2–4 skipping event. We quantified the exons 2–4 skipping in a panel of cell lines, and the results showed that total delta 2–4 (including both Delta 2–4 and delta 2–4/delta 7–8) expression was higher than Delta 2–4 expression in the cell panel as expected and supporting the validity of our new assays (Fig. 1F). To add context, we measured exons 7/8 TERT (indicative of FL TERT mRNA). These data indicate that Delta 2–4 containing exons 7/8 was a small fraction of the total number of transcripts containing exons 7/8 (Fig. S1D). The tested iPSC cell line showed the highest total delta 2–4 and Delta 2–4 expression compared to the other cell lines, followed by A549, Calu-6, H1299, H920, and HBEC (primary human bronchial epithelial cells). The detection of total delta 2–4 or Delta 2–4 in HBECs was very minimal, likely due to the very low overall TERT expression in normal primary cells. Based on our observations we concluded that lung cancer cells and telomerase positive iPSC (CHiPSC22) express a novel TERT isoform called Delta 2–4 that retains the original open reading frame and potentially codes for a new protein and that HBECs that lack telomerase activity do not express Delta 2–4 isoform making the Delta 2–4 isoform restricted to cells that express appreciable levels of TERT.

Next, we wanted to determine if TERT Delta 2–4 codes for a novel protein. Due to the low abundance of TERT protein which makes traditional western blotting (antibody based) protein detection challenging, we took several complimentary approaches to indirectly provide evidence of TERT Delta 2–4 protein coding capacity. Because previous reports have indicated that the majority of *TERT* mRNAs are sequestered in the nucleus, we considered the cellular localization of *TERT* delta 2–4 mRNAs. Additionally, our rationale was that RNA transcripts that could be translated into proteins, should have higher cytoplasmic to nuclear localization ratio than transcripts that lack protein coding capacity and are expected to be degraded by RNA decay mechanisms. We fractionated Calu-6 cells into nuclear and cytoplasmic fractions, extracted RNA from each fraction, and produced cDNAs with oligo dT priming (Fig. 1G, Fig. S1E). We measured MALAT1, which is a nuclear non-coding RNA (as a control for nuclear RNA fractionation validity), GAPDH (as a control for cytoplasmic RNA fractionation validity), total delta 2–4 (Delta 2–4 and delta 2–4/delta 7–8), Delta 2–4, potential FL (exons 7–8 containing *TERT*), minus beta (exons 7–8 excluding *TERT*), i11 (intron 11 detained *TERT*), and i14 (intron 14 detained *TERT*). We observed that most *TERT* mRNA variants (except i11 and i14) had significantly higher cytoplasmic localization ratio compared to MALAT1. When compared to GAPDH, which is a protein-coding gene, total delta 2–4, potential FL, minus beta, i11, and i14 *TERT* mRNA variants had significantly lower cytoplasmic localization ratios. As shown in Dumbović et al.^18^, intron 11 or 14 detained *TERT* transcripts were mostly localized in nucleus. Potential FL *TERT*, which is determined by measuring expression of *TERT* exons 7/8, had lower cytoplasmic localization ratio than GAPDH or Delta 2–4. This is likely because measured exons 7/8 containing *TERT* transcripts are not only the FL *TERT* mRNA, but also other non-full-length *TERT* mRNA variants the contain exons 7/8 (e.g., minus gamma, INS3, INS4, Del2, i11 or i14 detained *TERT* transcripts in nucleus). Total delta 2–4 includes both Delta 2–4 and delta 2–4/delta 7–8, and delta 2–4/delta 7–8 contains premature termination codon (PTC) in exon 10 induced by exons 7–8 splicing so it is expected to be degraded by non-sense mediated decay in the cytoplasm. Thus, we hypothesized that degradation of delta 2–4/delta 7–8 would result in lower cytoplasmic localization ratio of total delta 2–4 compared to Delta 2–4, and this was confirmed by our observations (Fig. 1G). Based on the observation that Delta 2–4 had a similar mRNA localization pattern to GAPDH, this provides evidence that Delta 2–4 potentially codes for a newly discovered TERT protein isoform.

### Impact of delta 2–4 on growth rate, telomere length and telomerase activity

**Fig. 2:**
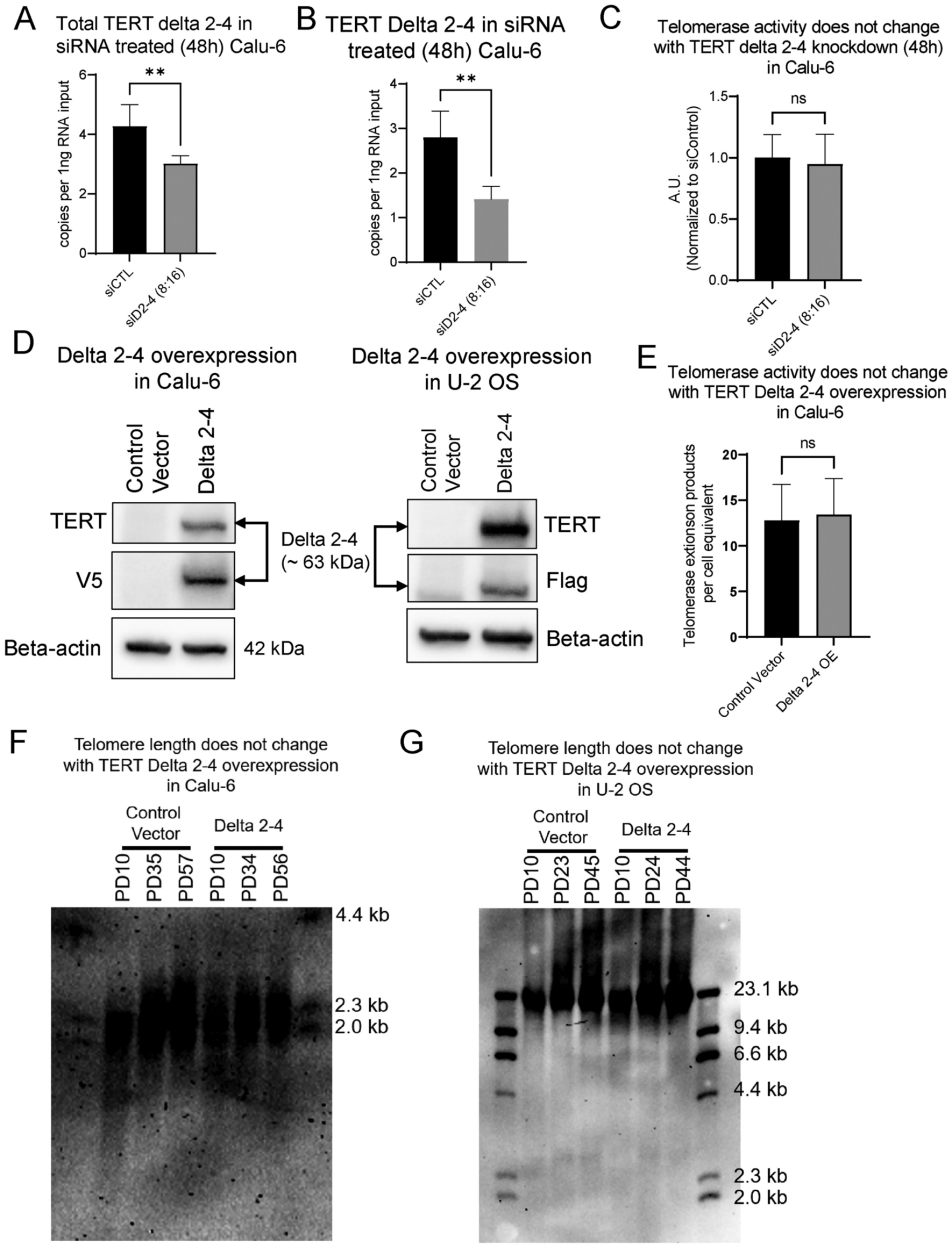
Impact of TERT Delta 2–4 isoform on growth rate, telomere length, and telomerase activity. (**A**) Total TERT delta 2–4 (Delta 2–4 and delta 2–4/delta 7–8) was measured following 48 h of siRNA treatment (determined by ddPCR; n = 6 biological replicates). (**B**) TERT Delta 2–4 expression level was measured following 48 h of siRNA treatment (determined by ddPCR; n = 6 biological replicates). (**C**) Telomerase activity did not change with TERT Delta 2–4 knockdown (48 h) in Calu-6 cells. Telomerase activity was determined by ddTRAP (n = 6 biological replicates per condition). (**D**) Flag-V5-Delta 2–4 expression was confirmed by TERT Y182 antibody, V5 antibody, and Flag antibody in Calu-6 cells (left) and U-2 OS cells (right). Beta-actin was used as a loading control. (**E**) Telomerase activity did not change with TERT Delta 2–4 overexpression in Calu-6 cells. Telomerase activity was determined by ddTRAP (n = 11 biological replicates per condition). (**F,G**) Telomere length did not change with TERT Delta 2–4 overexpression in Calu-6 (F) and U-2 OS (**G**) (determined by terminal restriction fragment (TRF) assay). Student’s t-test set at *p ≤ 0.05 for significance compared to siRNA control conditions. Data are presented as means ± standard deviations where applicable.

To further establish the potential function of TERT Delta 2–4, we performed loss of function studies using custom designed siRNAs. First, three siRNAs (siD2-4 (12:12), siD2-4 (8:16), and siD2-4 (16:8)) targeting different regions of *TERT* exons 1/5 junction and mixture of the same three siRNAs (siD2-4 (mix)) were tested (Fig. S2A). Our goal of this experiment was to find an siRNA that specifically reduces Delta 2–4 while leaving the remainder of *TERT* mRNA variants at or near steady state expression levels. Following 72 h of siRNA treatment, *TERT* Delta 2–4 expression was significantly reduced by siD2-4 (12:12) and siD2-4 (8:16) treatment compared to control (scrambled siRNA) treated Calu-6 cells (Fig. S2C). During the same treatment, total *TERT* delta 2–4 was not significantly reduced by any siRNAs (Fig. S2B). Since siRNAs are known to target RNAs in the cytoplasm better than RNAs in the nucleus^43^, we observed better knockdown of Delta 2–4 than we did of total *TERT* delta 2–4. This supports the idea that Delta 2–4 has a greater cytoplasmic localization of than delta 2–4/ delta 7–8 (Fig. 1G). The expression level of *TERT* transcripts containing exons 3–5 was measured to determine if total TERT transcripts without exons 1/5 junctions were reduced by the siRNAs targeting the exons 1/5 junction (i.e., targeting total TERT transcripts rather than specifically exon 1/5 junction containing transcripts). We found that siD2-4 (12:12) significantly reduced the expression level of *TERT* exons 3–5 containing transcripts indicating that this siRNA was not suitable for further study, while siD2-4 (8:16) did not reduce the expression level of *TERT* transcripts containing exons 3–5 (Fig. S2D). As a result, siD2-4 (8:16) was selected for further siRNA experiments. With 48 h of siRNA treatment (siD2-4 (8:16)) in Calu-6 cells, we observed a statistically significant 50% reduction of Delta 2–4 and a 25% reduction of total delta 2–4 (Delta 2–4 and delta 2–4/delta 7–8), suggesting that siRNA targeted mostly Delta 2–4 rather than delta 2–4/delta 7–8, transcripts in comparison to control treated Calu-6 cells (Fig. 2A,B, Fig. S2E). Despite the 50% reduction of *TERT* Delta 2–4 expression, telomerase activity, growth rate, and cell viability did not change after 48 h of siD2-4 (8:16) treatment compared to control (Fig. 2C, Fig. S2F,G). These data suggest that Delta 2–4 is not necessary for telomerase activity or for cell proliferation when other TERT isoforms are expressed. However, it should be noted that the residual Delta 2–4 could be contributing to our measured phenotypes.

We next tested gain of function by expressing exogenous Delta 2–4. We performed gain of function studies by establishing TERT Delta 2–4 expressing cell lines. We generated a codon optimized Delta 2–4 construct that was also siRNA resistant for rescue experiments. To avoid endogenous TERT antibody issues^44^, we added two N-terminal tags, FLAG and V5 tags allowing for reliable detection of the exogenously expressed Delta 2–4 protein (Fig. S2H). N-terminal tags do not impact TERT function as shown in previous studies^45–48^. Stable cell lines were successfully established in two different cell types (Calu-6 and U-2 OS) and expression of ectopic TERT Delta 2–4 was confirmed by antibodies targeting C-terminus of TERT (TERT Y182 antibody) and tags (FLAG and V5 antibodies) (Fig. 2D). The use of these two cells lines allowed us to test telomerase dependent phenotypes (Calu-6) and telomerase independent phenotypes (U-2 OS). Overexpression of TERT Delta 2–4 did not affect telomerase activity in Calu-6 cells, and telomerase activity was not detected from TERT Delta 2–4 expressing U-2 OS cells, as expected (Fig. 2E, Fig. S2I). In addition, expression of TERT Delta 2–4 did not change growth rate for over 50 days for both Calu-6 or U-2 OS, despite 50 and 30 population doublings (PDs), respectively, (Fig. S2J). Telomere length did not change in either cell line during the growth period as determined by terminal restriction fragment (TRF) analysis (Fig. 2F,G). To determine the cellular localization of Delta 2–4 we used immunocytochemistry in overexpression cells. These data indicate the Delta 2–4 is mainly observed in the nucleus of unstressed cells (Fig. S2K,L). Overall, based on the gain of function approaches showed that TERT Delta 2–4 functions are likely not related to telomerase activity, growth rate, or telomere length.

### Impact of Delta 2–4 on clonogenicity and resistance to cisplatin

**Fig. 3:**
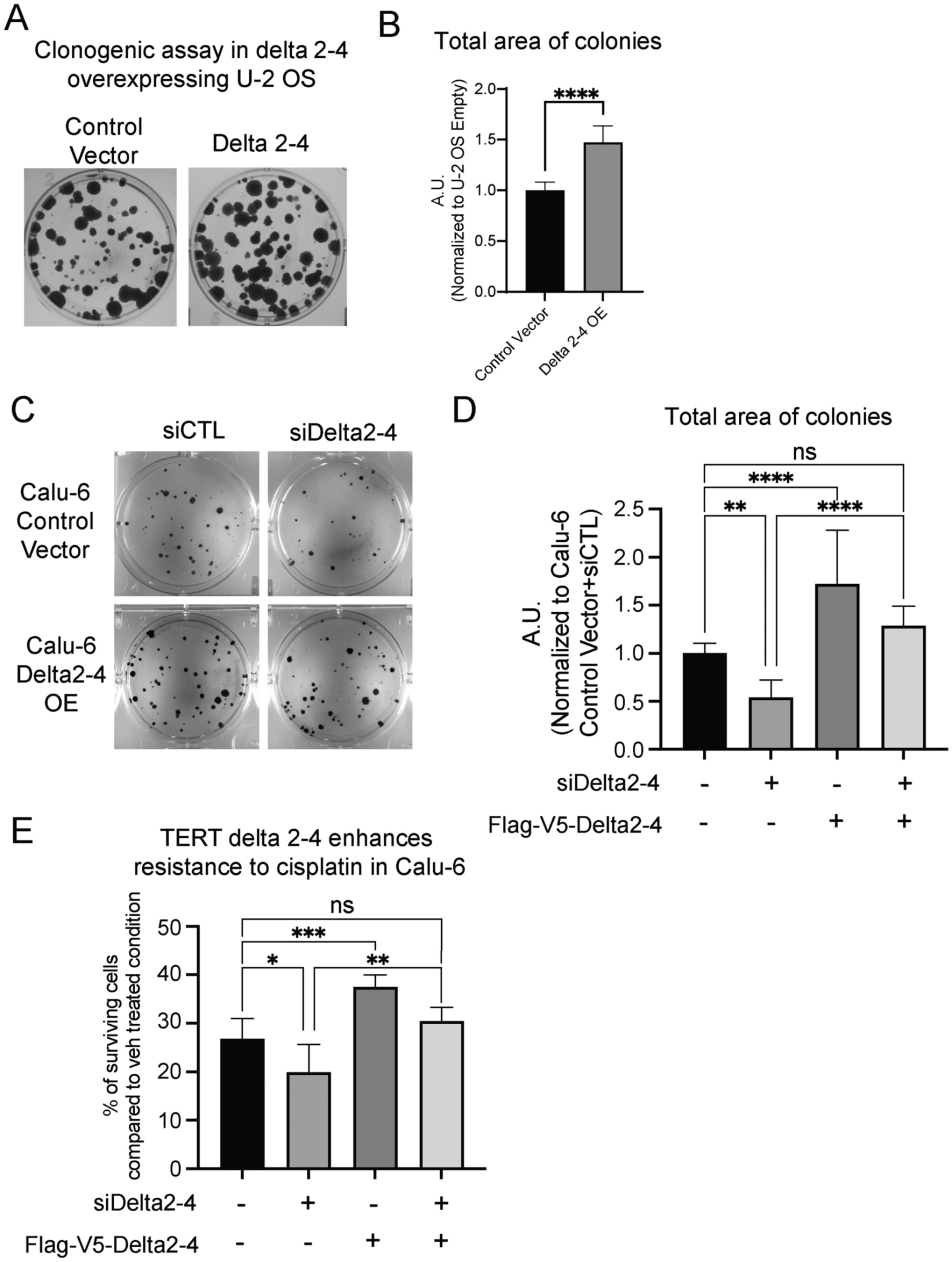
Impact of TERT Delta 2–4 isoform on clonogenicity and resistance to cisplatin. (**A,B**) Overexpression Delta 2–4 in telomerase deficient cell U-2 OS enhanced clonogenicity. Representative image (**A**) and quantification (**B**) are shown. (**C,D**) Knockdown of TERT Delta 2–4 reduced clonogenicity in telomerase positive cells Calu-6, whereas overexpression of TERT Delta 2–4 enhanced clonogenicity in Calu-6. Knockdown effect was rescued by siRNA resistant Delta 2–4 overexpression. Representative image (**C**) and quantification (**D**) are shown. (**E**) Calu-6 cells became more sensitive to cisplatin treatment (1.59 ug/mL, 48 h) by TERT Delta 2–4 knockdown and more resistant by TERT Delta 2–4 overexpression. Knockdown effect was rescued by siRNA resistant Delta 2–4 overexpression. For multiple group comparisons, *p*-value was calculated by Tukey’s multiple comparisons test following one-way ANOVA (ns > 0.05, *p < 0.05, **p < 0.01, ***p < 0.001, ****p < 0.0001). Data are presented as means ± standard deviations where applicable.

To continue to explore the functional roles of the TERT Delta 2–4 isoform, we hypothesized that TERT Delta 2–4 could be functioning to protect cells in some capacity^49,50^. We hypothesized that TERT Delta 2–4 would make cells more resistant to clonal density induced cell death. We used a classic assay of clonal density cell death resistance, the clonogenic assay, to test our hypothesis. In fact, we observed that expression of TERT Delta 2–4 enhanced clonogenicity (47% increased total area; 30% increased average size; 16% increased number of colonies) in comparison to empty vector expressing U-2 OS cells (Total area of colonies = Average size of colonies × Number of colonies). This observation supports the idea that the enhancement of clonogenicity by Delta 2–4 overexpression is telomerase independent (Fig. 3A,B, Fig. S3A,B). When TERT Delta 2–4 is expressed in Calu-6 cells, clonogenicity was also enhanced (72% increased total area; 42% increased average size; 18% increased number of colonies) compared to empty vector expressing cells. In addition, siRNA targeting TERT Delta 2–4 (siDelta2-4) treatment reduced clonogenicity (46% decreased total area; 18% decreased average size; 36% decreased number of colonies) and it was rescued by siRNA resistant Delta 2–4 expression supporting that the reduced clonogenicity by siRNA targeting TERT Delta 2–4 was on-target (Fig. 3C,D, Fig. S3C–E).

In a follow up experiment, we tested the impact of cisplatin, a common lung cancer chemotherapy, on our gain of function cells. Cisplatin, which is a platinum-based chemotherapeutic, binds to DNA and induces DNA damage resulting in death of cells^51^. Thus, we hypothesized that overexpression of TERT Delta 2–4 would enhance the cells’ resistance to cisplatin treatment in comparison to control. We performed dose response curves with cisplatin in TERT Delta 2–4 expressing U-2 OS and Calu-6 cells to determine EC50 values. We observed EC50 values of 0.72 μg/mL (0.54–0.89) and 1.2 μg/mL (0.95–1.5) for Calu-6 control compared to Delta 2–4 cells, respectively. This was a significant increase in the EC50 value for the Delta 2–4 cells compared to the controls (*p* = 0.002) indicating that the Delta 2–4 cells are more resistant to cisplatin compared to controls, supporting our hypothesis (Fig. S3F). However, expression of TERT Delta 2–4 in U-2 OS cells did not result in a significantly enhanced EC50 of cisplatin compared to control U-2 OS cells, (U-2 OS Delta 2–4 4.0 μg/mL (3.2–11.35) vs. control 3.5 μg/mL (3.0–4.8); S3G). In a rescue experiment, Calu-6 cells with TERT Delta 2–4 knockdown were 26% more sensitive to cisplatin treatment, whereas cells with ectopic expression of TERT Delta 2–4 were 40% more resistant to cisplatin treatment (Fig. 3E). Moreover, reduced resistance to cisplatin by siRNA knockdown was rescued by siRNA resistant TERT Delta 2–4 expression, supporting that the effect of siRNA knockdown on reduced resistance to cisplatin was an on-target effect of siRNA treatment. Overall, reduced clonogenicity and resistance to cisplatin treatment by TERT Delta 2–4 knockdown and increased clonogenicity and resistance to cisplatin treatment by TERT Delta 2–4 overexpression support our hypothesis: TERT Delta 2–4 enhances cells’ resistance to cell death.

### RNA sequencing reveals the impact of Delta 2–4 knockdown on the Calu-6 transcriptome

**Fig. 4:**
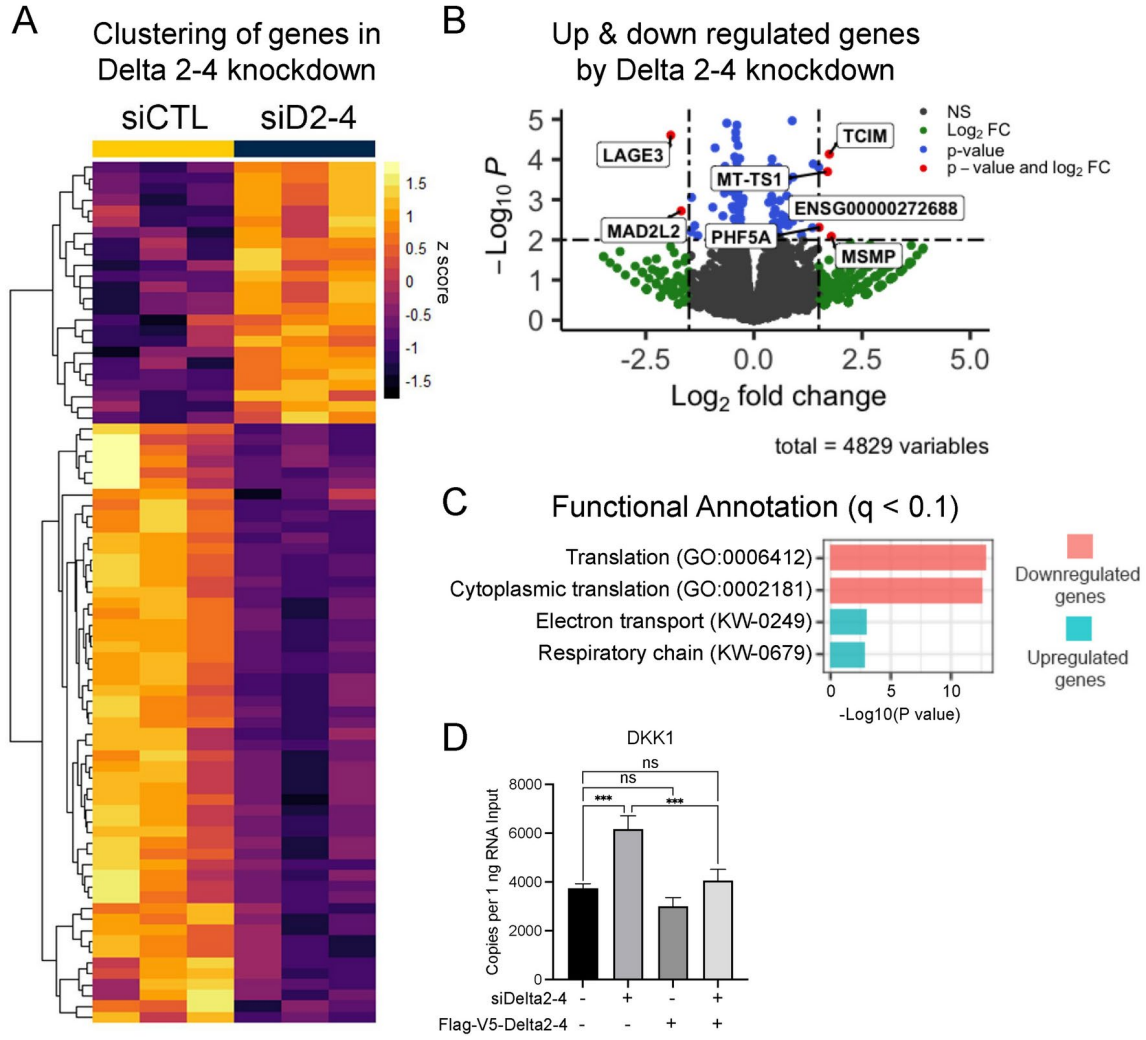
Transcriptomics analyses following TERT Delta 2–4 knock down in Calu-6 cells. (**A**) A heatmap visualizing clusters of differentially expressed genes (q < 0.1; 24 upregulated genes and 55 downregulated genes) in TERT Delta 2–4 knocked down Calu-6 cells. (**B**) Visualization of differentially expressed genes by volcano plot showing -log(p valule) and LFC (log twofold change). Dashed lines are at p value = 0.01 and LFC = 1.5 or − 1.5. Significantly differentially expressed seven genes are indicated (p < 0.01 and LFC <  − 1.5 or > 1.5). (**C**) Four significantly (q < 0.1) enriched biological processes by TERT Delta 2–4 were revealed by gene ontology analysis using differentially expressed genes. Two biological processes in red colors (cytoplasmic translation and translation) are related to downregulated genes, and the other two biological processes in blue colors (respiratory chain and electron transport) are related to upregulated genes with Delta 2–4 knockdown. (**D**) Upregulated DKK1 expression by TERT Delta 2–4 knockdown was rescued by siRNA resistant Delta 2–4 overexpression (determined by ddPCR; n = 3 biological replicates) in Calu-6 cells. For multiple group comparisons, *p*-value was calculated by Šídák's multiple comparisons test following one-way ANOVA (ns > 0.05, ***p < 0.001). Data are presented as means ± standard deviations where applicable.

To further understand the functions of Delta 2–4, we knocked down Delta 2–4 with siRNAs and performed RNA sequencing in Calu-6 cells. Hierarchical clustering based on Euclidean distance confirmed the clustering of biological replicates (Fig. S4A). Differential analysis identified significantly differentially expressed genes (q < 0.1; 24 upregulated genes and 55 downregulated genes) by Delta 2–4 knockdown (Fig. 4A). Next, we generated a volcano plot showing differentially expressed gene using the p-value and log2 fold change (LFC) values (*p* value < 0.01 and LFC <  − 1.5 or > 1.5; Fig. 4B). The top upregulated genes were TCIM (transcriptional and immune response regulator), MT-TS1 (mitochondrially encoded tRNA serine 1), ENSG00000272688 (long non-coding RNA gene), PHF5A (splicing factor; PHD finger protein 5A) and MSMP (microseminoprotein, prostate associated), and the top downregulated genes were LAGE3 (L Antigen Family Member 3) and MAD2L2 (mitotic arrest deficient 2 like 2). After identifying the top up and down regulated genes by Delta 2–4 knockdown, gene set enrichment analysis was performed using significantly differentially expressed genes. From the set of 55 significantly down regulated genes or 24 significantly upregulated genes, enriched biological processes (q < 0.1) were related to translation (down regulated genes, red boxes in Fig. 4C) or production of energy in mitochondria (upregulated genes, blue boxes in Fig. 4C), respectively. When RNA-Seq results from Flag-V5-Delta 2–4 expressing Calu-6 cells were incorporated, we found that changes in expression by TERT Delta 2–4 knockdown were restored by the rescue in 13 genes (p < 0.05 and opposite LFC direction; 6 upregulated and 7 downregulated; Fig. S4B). To further ensure that our results of the RNA sequencing were indeed related the Delta 2–4 knockdown and not due to off target siRNA effects we used miRDB^52^ to predict siRNA/miRNA targets of our Delta 2–4 siRNA (5ʹ-UCCGCCAGGCCGAGCGUCUCACCU). Among the 37 predicted targets, no target genes were found among our 79 differentially expressed genes by TERT Delta 2–4 knockdown (Fig. S4C). DKK1 (Dickkopf-1) expression was upregulated by TERT Delta 2–4 knockdown and the upregulation was attenuated by siRNA resistant TERT Delta 2–4 overexpression (Fig. 4D), as validated by RT-ddPCR (reverse transcription- droplet digital PCR).

## Discussion

Increased telomerase activity is associated with greater than 85% of human cancers, including lung cancers^53^. Significant advances have been made in understanding of telomerase composition, biogenesis, trafficking, and regulation. However, fundamental questions remain about the function of alternative splicing isoforms of TERT. Here we report a catalogue of *TERT* mRNA variants from four cells lines. We focused on a novel TERT isoform that we called TERT Delta 2–4. Based on our experimental data we determined that Delta 2–4 does not function in telomere biology but ascribed its function to protecting cells from cell death, likely via translational promotion and mitochondrial protection. Combine our data indicate that the major function of the extensive alternative splicing of TERT pre-mRNAs is to control the abundance of FL TERT and telomerase activity, while low abundant isoforms such as Delta 2–4 likely play supporting roles potentially in translation and mitochondria of rapidly dividing cells such as embryonic stem cells and cancer cells. Additional studies will be needed to determine if any of these identified isoforms are therapeutically targetable.

Previous reports have identified TERT alternative splicing isoforms from a variety of cell types^8,16,17,54,55^. These studies utilized either traditional cDNA cloning followed by Sanger sequencing to identify AS variants or short read RNA sequencing followed by mathematical reconstruction of isoforms based on overlapping reads and probability modeling. While these methods are valid and have identified some transcript variants, newer and more advanced sequencing modalities allow for richer investigation of the transcript landscape of TERT in cells. To directly investigate full length cDNA molecules which might contain multiple splicing events in combination, we chose to use single molecule long read sequencing. This technology provides unambiguous identification of mRNA transcript variants and avoids potential bias of short read sequencing in the mathematical reconstruction of transcript variants. *TERT* is a low abundant gene that is expressed at 1–40 transcript copies per cell^56^. To ensure robust detection of *TERT* we prepared *TERT* specific sequencing libraries using PCR with primers targeting exons 1 and 16. We discovered 32 *TERT* mRNA transcripts that have not been reported previously to our knowledge. We observed several transcript variants of *TERT* that were previously known as well. Our main conclusion from the long-read RNA sequencing data is that *TERT* is extensively alternatively spliced in telomerase positive cells. We hypothesize that the extensive splicing of TERT to many non-coding or nuclear retained transcripts is to regulate the abundance of FL *TERT* mRNA in the cytoplasm that can be translated into FL TERT proteins. We extend this abundance regulating idea of TERT alternative splicing to include the regulation of the function of TERT as we identified several novel protein coding transcripts that do not appear to be dominant-negative TERTs, retained in the nucleus, or degraded by nonsense-mediated mRNA decay. An alternative hypothesis of the extensive alternative splicing of the TERT locus transcript (pre-mRNA) is that it arises from molecular error and that the resulting products are biological noise^57^. Either way, this mechanism, purposeful or error, regulates the abundance of FL TERT. Of the few novel protein coding transcripts we identified; we pursued determining the function of TERT Delta 2–4.

We focused on TERT Delta 2–4 because of its abundance and potential to code for a new protein isoform with potentially new functions in cell biology. Exons 2 to 4 are translated into the telomerase RNA template binding domain (TRBD in Fig. 1A) and the complete skipping of these exons does not induce frameshifting or a premature stop codon. We showed that Calu-6 cells with Delta 2–4 reduction were 26 percent more sensitive to cisplatin treatment (Fig. 3E). In addition, overexpression of Delta 2–4 TERT in Calu-6 made the cells more resistant to cisplatin treatment (Fig. S3F). Our experiments point out that additional protein coding isoforms of TERT may exist and that further experimentation is required to develop detailed functional annotation of these isoforms.

To further investigate the functional role of Delta 2–4 TERT, RNA sequencing was performed following Delta 2–4 knockdown in Calu-6 cells. Gene ontology analysis revealed that genes involved in translation were downregulated while genes involved in mitochondrial energy production were upregulated following reduction in Delta 2–4 TERT. The top down-regulated genes related to translation included MRPL11, MRPL20, RPL10, RPL13A, RPL28, RPL32, RPL35, RPL36, RPL38, RPS15, RPS2, and RPS21. The top up regulated genes related to mitochondrial function included CYCS, MT-ND1, MT-CYB, and MT-CO2. Our rescue and validation data revealed DKK1, an inhibitor of the classic WNT signaling pathway^57^. TERT and TERT isoforms (Delta 4–13^17^), have been implicated in WNT signaling and our data add to this growing body of literature. Further experiments will be needed to determine the nature of this finding. While our experiments cannot determine direct or indirect effects of Delta 2–4 knockdown, we conclude that delta 2–4 impacts cells likely at the levels of protein translation, WNT signaling, and energy (ATP) production.

Our study is not without limitations. First, when we prepared the TERT specific sequencing library, we used PCR which may have skewed the expression levels shown in the heatmap or generated artifactual sequences (Fig. S1A). Thus, validation of TERT transcripts must be performed before functional studies of novel TERT isoforms are completed. For instance, we confirmed expression of *TERT* Delta 2–4 mRNA by several methods including cDNA cloning, sanger sequencing of the clones, and ddPCR assays of the endogenous transcripts. Another limitation is that *TERT* transcripts that do not contain exons 1 or 16 were not included in our list of isoforms because we enrichment for transcripts that contained exons 1 and 16 by PCR. Also, we did not detect transcripts that were longer than 4-kilobases due to our library preparation and size exclusion. However, we were looking for mRNA variants that retained the original open reading frame and coded for proteins, thus this was a known and purposeful bias in our analysis. Although we discovered many TERT isoforms in induced pluripotent stem cells and three non-small cell lung cancer cells, further discovery may be made by using different types of cells, such as embryonic stem cells, somatic cells, adult stem cells, and cells from other types of cancers, as well as tissues from humans. Finally, we were not able to show endogenous protein expression of Delta 2–4 isoform due to the low expression and lack of robust delta 2–4 TERT antibodies^44^. Another limitation to our loss of function data is the fact that we only achieved a 50% knockdown of the mRNA leaving residual Delta 2–4 that could be functioning in telomere maintenance pathways and cell proliferation and survival. Further research should explore more potent loss of function technologies such as CRISPR/Cas13 mRNA depletion or better interfering RNAs. In terms of the overexpression model, we observed high levels of delta 2–4 mRNA and tagged protein expression that is higher than physiological levels of Delta 2–4, therefore our data should be interpreted cautiously with potential hypermorphic impacts of the expressed protein. Finally, we did not test the localization of Delta 2–4 following cellular stressors such as cisplatin treatment which could be informative to the potential function of Delta 2–4 TERT; future research would benefit from these studies.

In conclusion, we show that TERT is extensively alternatively spliced to regulate FL TERT expression and that a novel isoform, Delta 2–4, likely has functions outside of telomere synthesis. We provide evidence that Delta 2–4 functions are related to protein translation and mitochondrial function which act to enhance the clonogenicity of cells and to make lung cancer cells more resistant to cisplatin treatment. Implications of our data are that additional TERT isoforms may code for novel proteins and that better cancer drug efficiency could be achieved by targeting TERT Delta 2–4.

## Supplementary Information

Supplementary Information 1.

Supplementary Information 2.

## Data Availability

All raw files used to generate figures can be found in Deep blue data repository (10.7302/hm4z-r975). High throughput sequencing data can be found at Gene expression omnibus (GEO) at GSE279325 (long read TERT specific) and GSE279438 (TERT Delta 2–4 siRNA full transcriptome in Calu-6 cells).
