# Supplementary material for: Discovery and characterization of a novel telomerase alternative splicing isoform that protects lung cancer cells from chemotherapy induced cell death
Source: Sci Rep. 2025 Feb 25;15:6787. doi: 10.1038/s41598-025-90639-3 (PMC11861669; doi:10.1038/s41598-025-90639-3)
Supplement: Supplementary file 1 — Supplementary Information 1. [file 41598_2025_90639_MOESM1_ESM.pdf]

Figure S1A

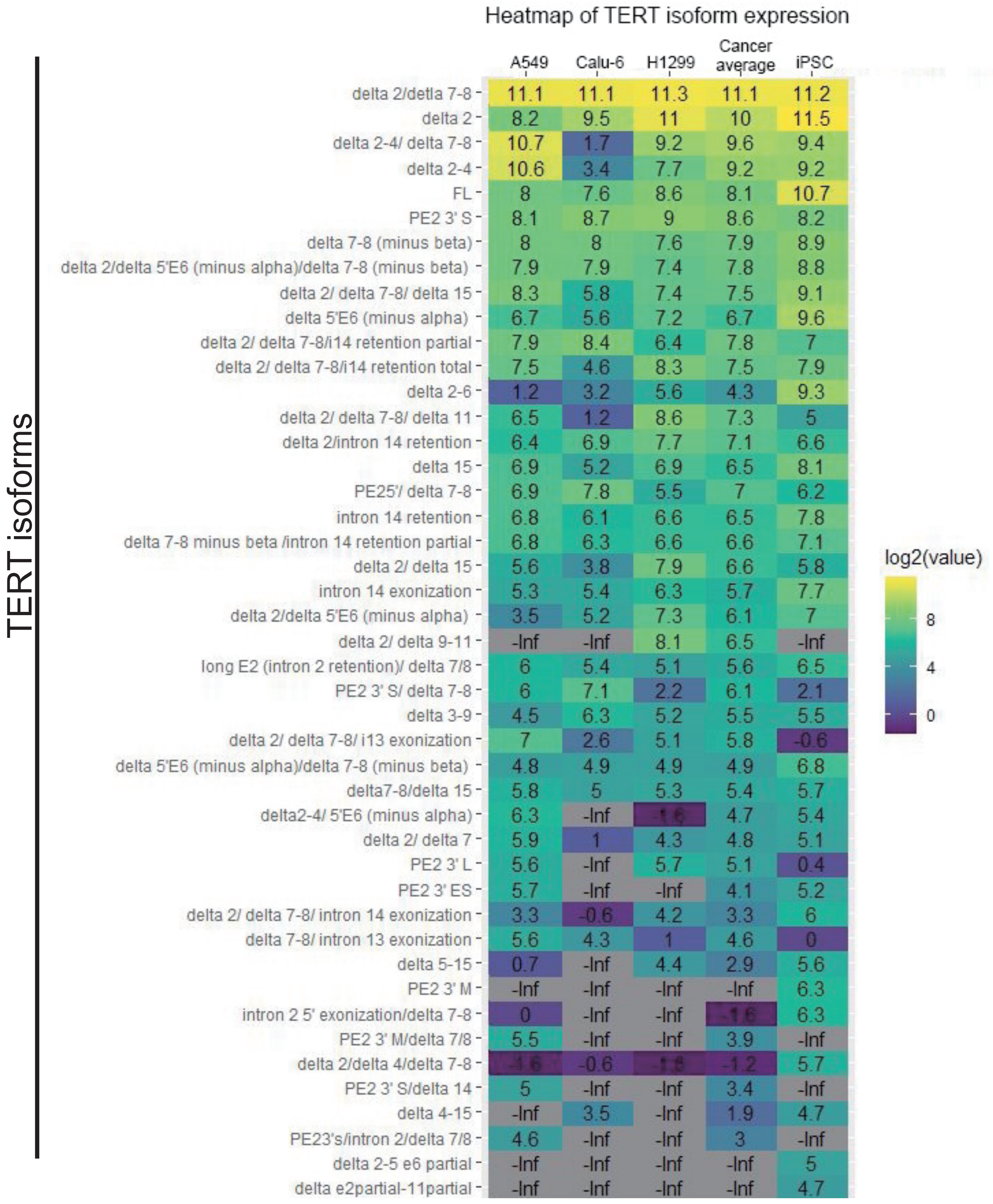
Heatmap of TERT isoform expression
A549 Calu-6 H1299 Cancer average iPSC
TERT isoforms
log2(value)
8
4
0
delta 2/detla 7-8 11.1 11.1 11.3 11.1 11.2
delta 2 8.2 9.5 11 10 11.5
delta 2-4/ delta 7-8 10.7 1.7 9.2 9.6 9.4
delta 2-4 10.6 3.4 7.7 9.2 9.2
FL 8 7.6 8.6 8.1 10.7
PE2 3' S 8.1 8.7 9 8.6 8.2
delta 7-8 (minus beta) 8 8 7.6 7.9 8.9
delta 2/delta 5'E6 (minus alpha)/delta 7-8 (minus beta) 7.9 7.9 7.4 7.8 8.8
delta 2/ delta 7-8/ delta 15 8.3 5.8 7.4 7.5 9.1
delta 5'E6 (minus alpha) 6.7 5.6 7.2 6.7 9.6
delta 2/ delta 7-8/i14 retention partial 7.9 8.4 6.4 7.8 7
delta 2/ delta 7-8/i14 retention total 7.5 4.6 8.3 7.5 7.9
delta 2-6 1.2 3.2 5.6 4.3 9.3
delta 2/ delta 7-8/ delta 11 6.5 1.2 8.6 7.3 5
delta 2/intron 14 retention 6.4 6.9 7.7 7.1 6.6
delta 15 6.9 5.2 6.9 6.5 8.1
PE25'/ delta 7-8 6.9 7.8 5.5 7 6.2
intron 14 retention 6.8 6.1 6.6 6.5 7.8
delta 7-8 minus beta /intron 14 retention partial 6.8 6.3 6.6 6.6 7.1
delta 2/ delta 15 5.6 3.8 7.9 6.6 5.8
intron 14 exonization 5.3 5.4 6.3 5.7 7.7
delta 2/delta 5'E6 (minus alpha) 3.5 5.2 7.3 6.1 7
delta 2/ delta 9-11 -Inf -Inf 8.1 6.5 -Inf
long E2 (intron 2 retention)/ delta 7/8 6 5.4 5.1 5.6 6.5
PE2 3' S/ delta 7-8 6 7.1 2.2 6.1 2.1
delta 3-9 4.5 6.3 5.2 5.5 5.5
delta 2/ delta 7-8/ i13 exonization 7 2.6 5.1 5.8 -0.6
delta 5'E6 (minus alpha)/delta 7-8 (minus beta) 4.8 4.9 4.9 4.9 6.8
delta7-8/delta 15 5.8 5 5.3 5.4 5.7
delta2-4/ 5'E6 (minus alpha) 6.3 -Inf -1.6 4.7 5.4
delta 2/ delta 7 5.9 1 4.3 4.8 5.1
PE2 3' L 5.6 -Inf 5.7 5.1 0.4
PE2 3' ES 5.7 -Inf -Inf 4.1 5.2
delta 2/ delta 7-8/ intron 14 exonization 3.3 -0.6 4.2 3.3 6
delta 7-8/ intron 13 exonization 5.6 4.3 1 4.6 0
delta 5-15 0.7 -Inf 4.4 2.9 5.6
PE2 3' M -Inf -Inf -Inf -Inf 6.3
intron 2 5' exonization/delta 7-8 0 -Inf -Inf -1.6 6.3
PE2 3' M/delta 7/8 5.5 -Inf -Inf 3.9 -Inf
delta 2/delta 4/delta 7-8 -1.6 -0.6 -1.8 -1.2 5.7
PE2 3' S/delta 14 5 -Inf -Inf 3.4 -Inf
delta 4-15 -Inf 3.5 -Inf 1.9 4.7
PE23's/intron 2/delta 7/8 4.6 -Inf -Inf 3 -Inf
delta 2-5 e6 partial -Inf -Inf -Inf -Inf 5
delta e2partial-11partial -Inf -Inf -Inf -Inf 4.7

Figure S1B

Transcript Name	ORF	Premature stop codon	Novelty	Transcript model
delta 2; delta 7-8	291 bp	In exon 3	Known	
delta 2	291 bp	In exon 3	Known (Del2)	
delta 2-4; delta 7-8	693 bp	In exon 10	Novel	
delta 2-4	1671 bp	In-frame	Novel	
FL	3405 bp	In-frame	Known	
PE2 3' S	636 bp	In exon 3	Novel	
delta 7-8	2013 bp	In exon 5	Known (-β)	
delta 2; delta 5'E6; delta 7-8	291 bp	In exon 3	Known	
delta 2; delta 7-8; delta 15	291 bp	In exon 3	Novel	
delta 5'E6	3273 bp	In-frame	Known (-α)	
delta 2; delta 7-8; i14 ret part	291 bp	In exon 3	Novel	
delta 2; delta 7-8; i14 ret tot	291 bp	In exon 3	Novel	
delta 2-6	1368 bp	In-frame	Novel	
delta 2; delta 7-8; delta 11	291 bp	In exon 3	Known	
delta 2; i14 ret part	291 bp	In exon 3	Known	
delta 15	3261 bp	In-frame	Novel	
PE2 5'; delta 7-8	450 bp	In exon 3	Novel	
i14 retention	3291 bp	In retained intron 14	Novel	
delta 7-8; i14 ret part	2424 bp	In exon 10	Novel	
delta 2; delta 15	291 bp	In exon 3	Novel	
i14 exonization	3483 bp	In-frame	Known	
delta 2; delta 5'E6	291 bp	In exon 3	Known	
delta 2; delta 9-12	291 bp	In exon 3	Novel	
long E2 (i2 ret); delta 7-8	1725 bp	In retained intron 2	Known	
PE2 3'S; delta 7-8	264 bp	In exon 2	Novel	
delta 3-8	1725 bp	In exon 10	Novel	
delta 2; delta 7-8; i13 exon	291 bp	In exon 3	Novel	
delta 5'E6; delta 7-8	2388 bp	In exon 10	Known	
delta 7-8; delta 15	2424 bp	In exon 10	Novel	
delta 2-4; 5'E6	1650 bp	In-frame	Novel	
delta 2; delta 8	291 bp	In exon 3	Novel	
PE2 3'L	171 bp	In exon 2	Novel	
PE2 3' ES	2202 bp	In-frame	Novel	
delta 2; delta 7-8; i14 exon	291 bp	In exon 3	Known	
delta 7-8; i13 exon	2424 bp	In exon 10	Novel	
delta 5-15	1920 bp	In-frame	Novel	
PE2 3' M	279 bp	In exon 2	Novel	
i2 5' exon; delta 7-8	153 bp	In retained intron 2	Novel	
PE2 3'M; delta 7-8	828 bp	In exon 3	Novel	
delta 2; delta 4; delta 7-8	291 bp	In exon 3	Novel	
PE2 3'S; delta 15	594 bp	In exon 3	Novel	
delta 4-15	1806 bp	In-frame	Novel	
PE2 3'S; i2; delta 7-8	282 bp	In retained intron 2	Novel	
delta 2-5; E6 part	282 bp	In exon 8	Novel	
delta E2-E11 part	1632 bp	In-frame	Novel	

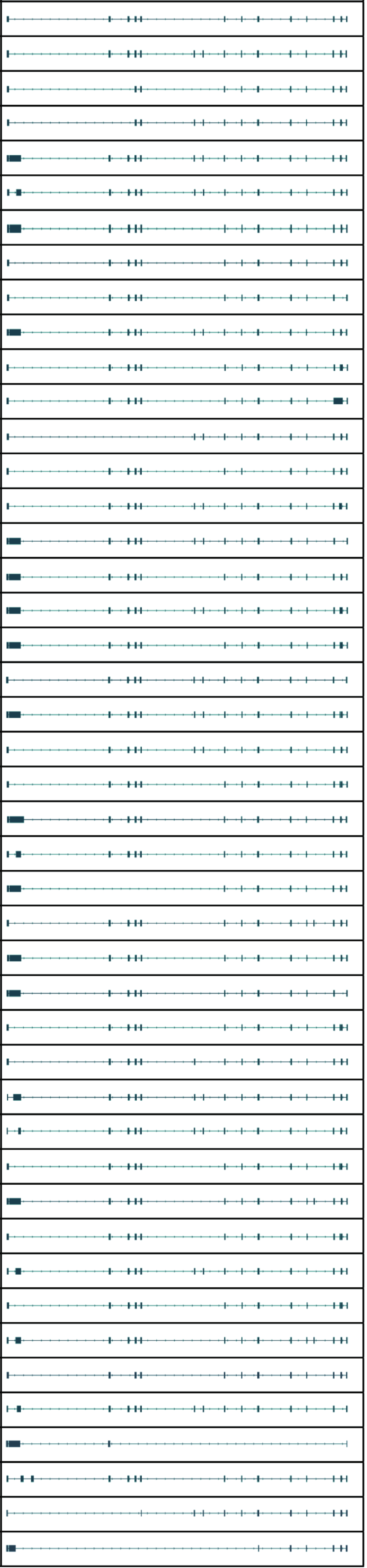

Figure S1

C

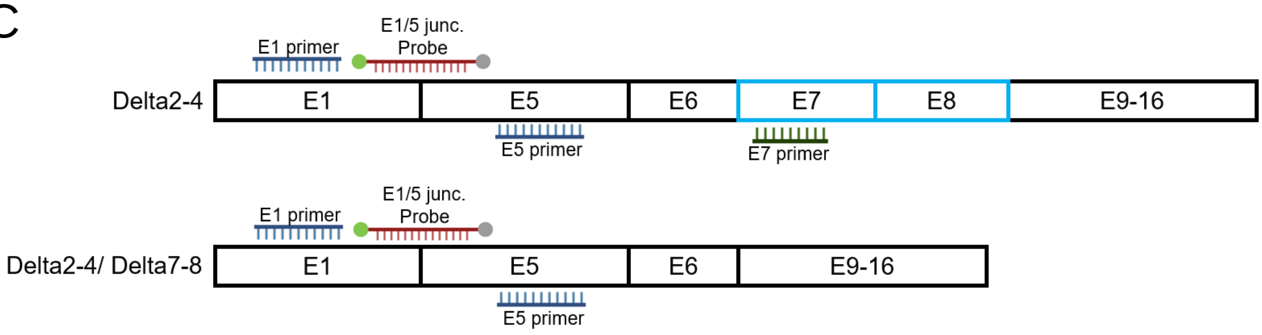

D

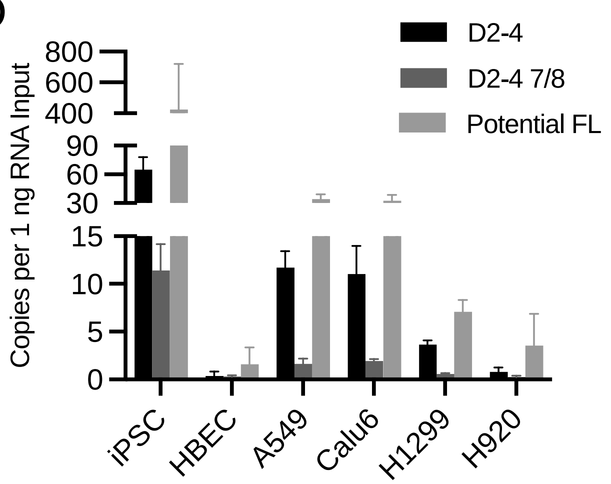

E

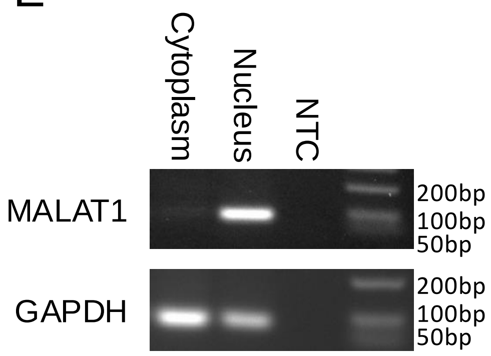

Supplementary Figure 1. **A**. Heatmap of TERT isoform log2 transformed expression levels of 45 TERT isoforms from A549, Calu-6, H1299, Cancer average (average of A549, Calu-6, and H1299), and iPSC. Alignment was based on expression level. **B**. Table showing transcript name, open reading frame (ORF), premature stop codon, novelty, and transcript model of the 45 TERT isoforms. Order matches to the heatmap. **C**. Primers and Probes for ddPCR targeting TERT Delta 2-4 with exons 7/8 (Delta 2-4; Top) or Delta 2-4 without exons 7/8 (Delta 2-4/ Delta 7-8; bottom). TERT exon numbers and targeted exons are indicated. Exon 1 and Exon 5 primers detects both Delta 2-4 and Delta2-4/ Delta 7-8 (total TERT Delta 2-4) whereas Exon1 and Exon 7 primers only targets TERT Delta 2-4. **D**. TERT Delta 2-4 and Potential FL (exons 7-8 including TERT transcripts) expression levels in cell panels (determined by ddPCR; n = 3 biological replicates per condition). Total delta 2-4 indicates both exons 2-4 skipping variants including Delta 2-4 and delta2-4/ delta 7-8, and Delta 2-4 indicates exons 2-4 skipping with exon 7/8 inclusion (note the capital D) (determined by ddPCR; n = 3 biological replicates per condition). **E**. Localization of nuclear non-coding RNA MALAT1 and protein-coding house-keeping gene GAPDH (Determined by RT-PCR followed by nuclear or cytoplasmic RNA extraction from Calu-6 cells; NTC: non-template control; n=3 biological replicates per condition; DNA Hi-Lo ladder on the right corner).

Figure S2

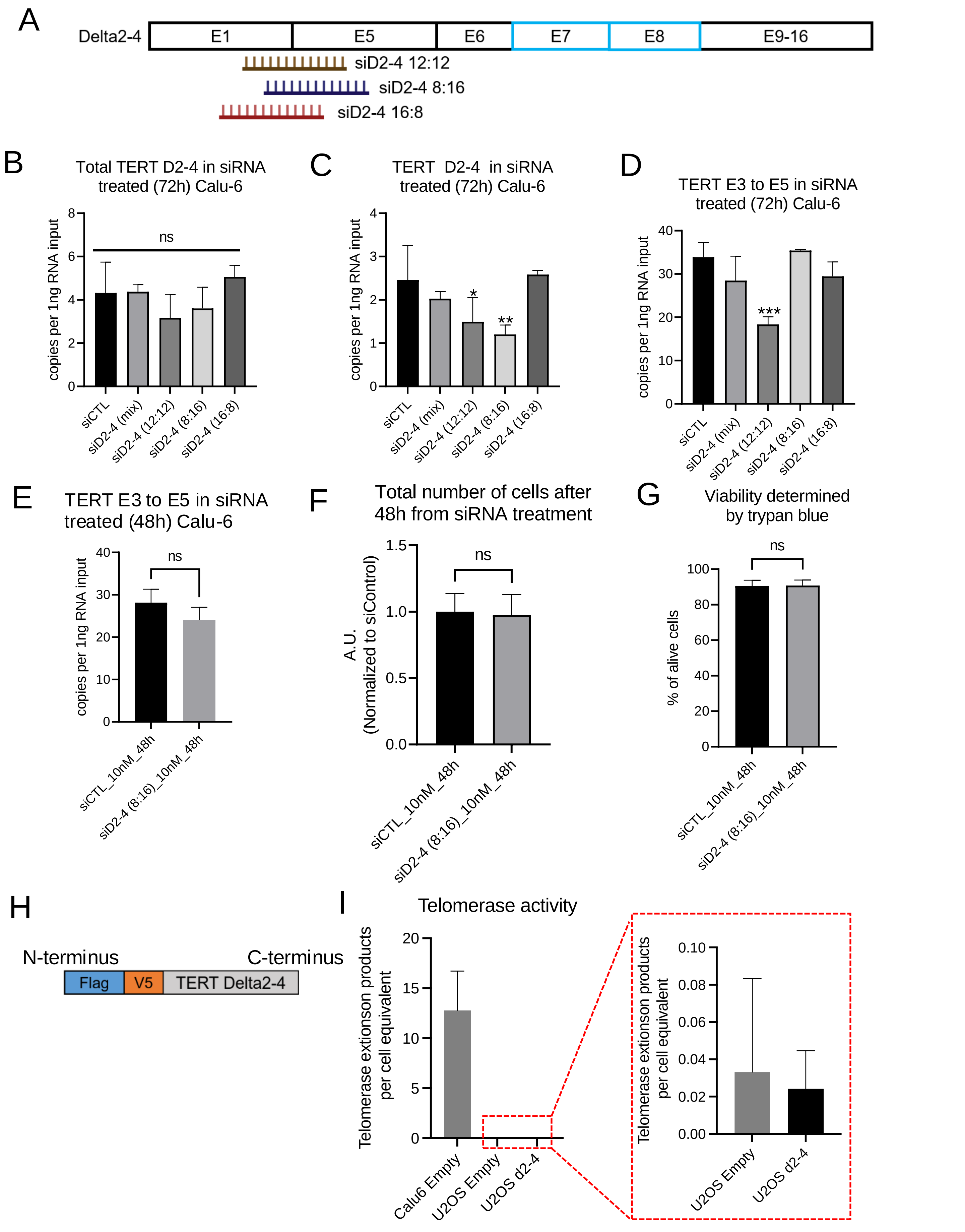
A
Delta2-4
E1
E5
E6
E7
E8
E9-16
siD2-4 12:12
siD2-4 8:16
siD2-4 16:8
B
Total TERT D2-4 in siRNA treated (72h) Calu-6
copies per 1ng RNA input
ns
siCTL
siD2-4 (mix)
siD2-4 (12:12)
siD2-4 (8:16)
siD2-4 (16:8)
C
TERT D2-4 in siRNA treated (72h) Calu-6
copies per 1ng RNA input
*
**
siCTL
siD2-4 (mix)
siD2-4 (12:12)
siD2-4 (8:16)
siD2-4 (16:8)
D
TERT E3 to E5 in siRNA treated (72h) Calu-6
copies per 1ng RNA input
***
siCTL
siD2-4 (mix)
siD2-4 (12:12)
siD2-4 (8:16)
siD2-4 (16:8)
E
TERT E3 to E5 in siRNA treated (48h) Calu-6
copies per 1ng RNA input
ns
siCTL_10nM_48h
siD2-4 (8:16)_10nM_48h
F
Total number of cells after 48h from siRNA treatment
A.U. (Normalized to siControl)
ns
siCTL_10nM_48h
siD2-4 (8:16)_10nM_48h
G
Viability determined by trypan blue
% of alive cells
ns
siCTL_10nM_48h
siD2-4 (8:16)_10nM_48h
H
N-terminus
C-terminus
Flag
V5
TERT Delta2-4
I
Telomerase activity
Telomerase extionson products per cell equivalent
Calu6 Empty
U2OS Empty
U2OS d2-4
Telomerase extionson products per cell equivalent
U2OS Empty
U2OS d2-4

Figure S2

J

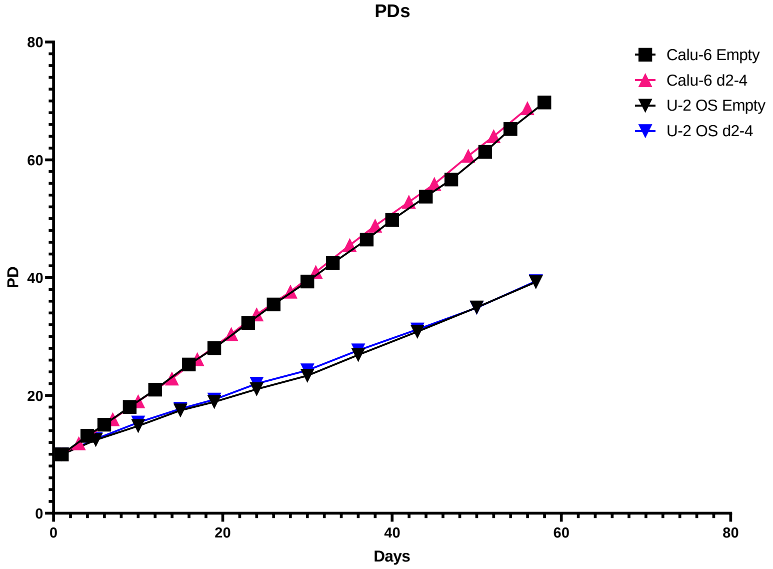

K

Immunostaining in U-2 OS cells

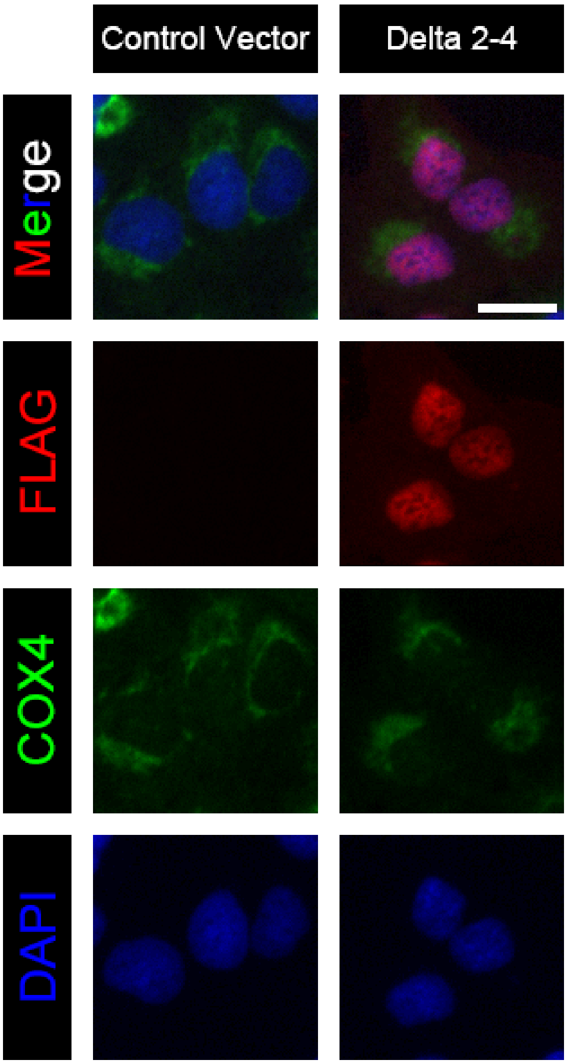

L

Immunostaining in Calu-6 cells

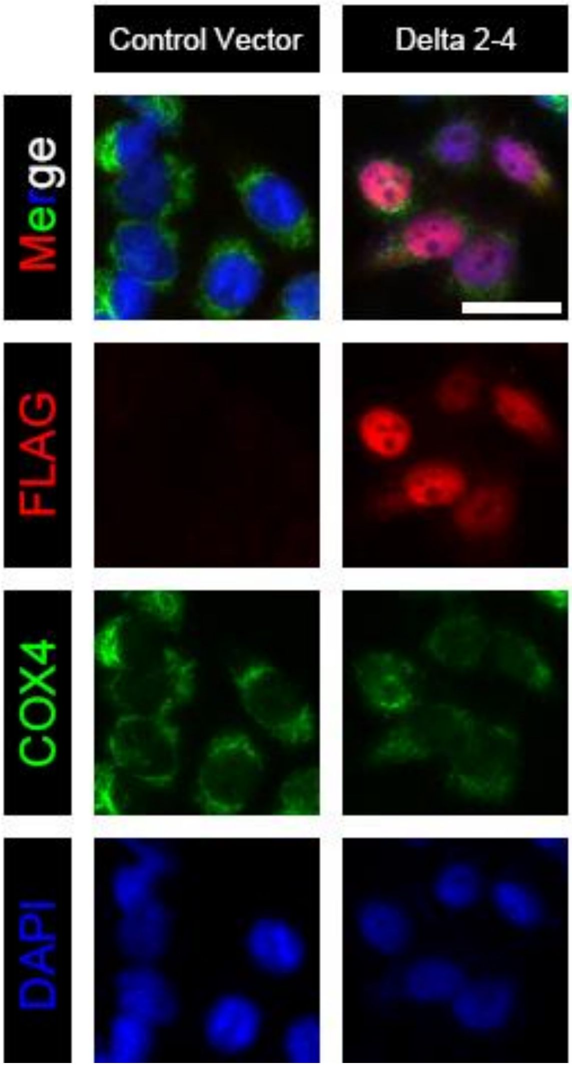

Supplementary Figure 2. **A**. Three siRNAs (D2-4 12:12: 12 exon 1 nucleotides and 12 exon 5 nucleotides; D2-4 8:16: 8 exon 1 nucleotides and 16 exon 5 nucleotides; D2-4 16:8: 16 exon 1 nucleotides and 8 exon 5 nucleotides) targeting exons 1/5 junction are depicted. **B**. Total TERT Delta 2-4 (Delta 2-4 and Delta 2-4/ Delta 7-8) expression level was measured following 72 hours after siRNA treatment (determined by ddPCR; n = 3 biological replicates per condition). **C**. TERT Delta 2-4 expression level was measured following 72 hours after siRNA treatment (determined by ddPCR; n = 3 biological replicates per condition). **D**. TERT transcript with exons 3 to 5 expression level was measured following 72 hours after siRNA treatment (determined by ddPCR; n = 3 biological replicates per condition). **E**. TERT transcript with exons 3 to 5 expression level was measured following 48 hours after siRNA treatment. **F** and **G**. Neither of total number of cells (F; Alive and dead cells) nor cell viability (G; determined by trypan blue) showed difference in calu-6 cells treated with siRNA for 48 hours targeting TERT Delta 2-4 (n = 6 biological replicates per condition). **H**. Cartoon of codon optimized Flag-V5-TERT Delta 2-4. **I**. Telomerase activity was not detected with TERT Delta 2-4 overexpression in U-2 OS cells. Telomerase activity was determined by ddTRAP (n = 3 biological replicates per condition). **J**. Population doubling did not change with Delta 2-4 overexpression in Calu-6 or U-2 OS cells (x-axis: number of days from completion of selection; y-axis: population doubling). (**K**-**L**). U-2 OS cells (K) and Calu-6 cells (L) were co-immunostained with antibodies specific to FLAG (red) and COX4 (green). DNA was visualized with DAPI (blue). Scale bars: 25 μm. Immunostaining revealed nuclear localization of N-terminus FLAG Delta 2-4.

Figure S3

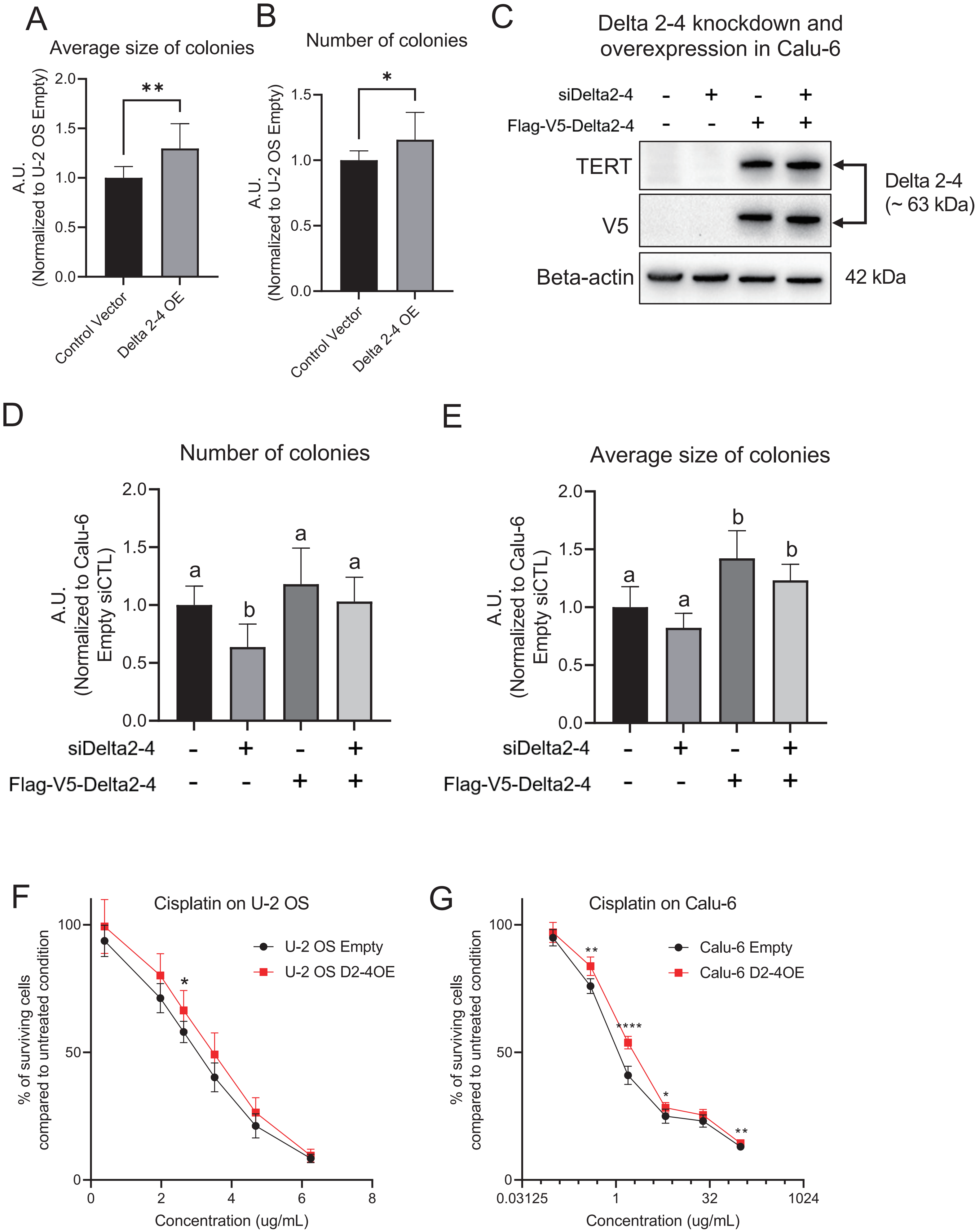
A
Average size of colonies
A.U.
(Normalized to U-2 OS Empty)
**
Control Vector
Delta 2-4 OE
B
Number of colonies
*
C
Delta 2-4 knockdown and overexpression in Calu-6
siDelta2-4
Flag-V5-Delta2-4
TERT
V5
Beta-actin
Delta 2-4 (~ 63 kDa)
42 kDa
D
Number of colonies
A.U.
(Normalized to Calu-6 Empty siCTL)
E
Average size of colonies
F
Cisplatin on U-2 OS
U-2 OS Empty
U-2 OS D2-4OE
% of surviving cells compared to untreated condition
Concentration (ug/mL)
G
Cisplatin on Calu-6
Calu-6 Empty
Calu-6 D2-4OE

Supplementary Figure 3. **A**. Average size of colonies with TERT Delta 2-4 overexpression in U-2 OS cells. **B**. Number of colonies with TERT Delta 2-4 overexpression in U-2 OS cells (n = 6 biological replicates per condition). **C**. Westernblot showing siD2-4 did not reduce Delta 2-4 expression. **D**. Number of colonies from control vector expressing calu-6 cells or TERT Delta 2-4 expressing calu-6 cells with 48 hours of siRNA control treatment or siRNA targeting TERT Delta 2-4 (n = 12 biological replicates per condition). **E**. Average size of colony from control vector expressing calu-6 cells or TERT Delta 2-4 expressing calu-6 cells with 48 hours of siRNA control treatment or siRNA targeting TERT Delta 2-4 (n = 12 biological replicates per condition). **F** and **G**. Cells with Delta 2-4 overexpression are more resistant to cisplatin treatment in U-2 OS (C) and Calu-6 (D) (x-axis: cisplatin concentration (ug/mL); y-axis: percentage of surviving cells compared to cisplatin untreated condition; n = 6 biological replicates per condition).

Figure S4

A

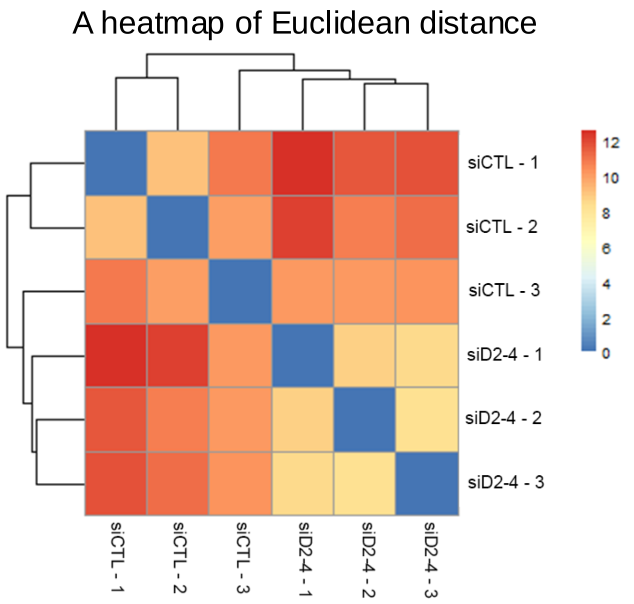

B

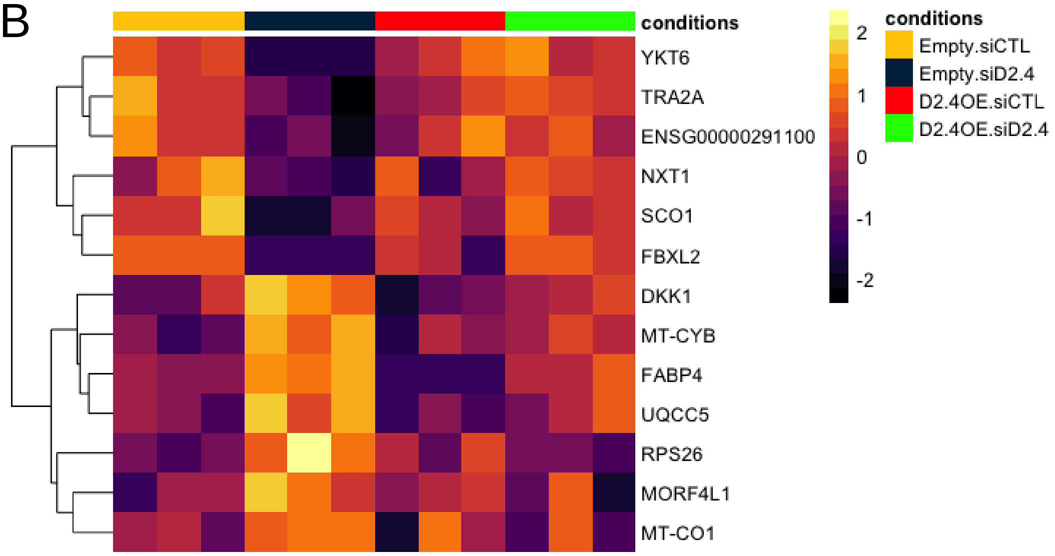

C

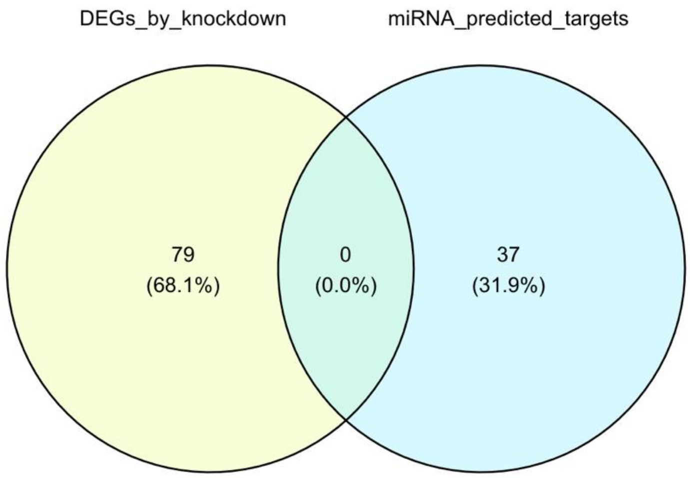

Supplementary Figure 4. **A**. Euclidean distance heatmap showing clustering of biological replicates. **B**. A heatmap showing 13 genes that are differentially expressed by TERT Delta 2-4 knockdown ($p < 0.05$; 6 downregulated genes and 7 upregulated genes) and rescued ($p < 0.05$ and Log fold change in the opposite direction) in Calu-6 cells with stable overexpression of TERT Delta 2-4. **C**. Overlap analysis (Venn diagram) between 79 differentially expressed genes and potential siDelta2-4 targets predicted by miRDB (37 genes). No overlapping target genes were found.

Supplementary information

Table S1. Primer and Probe sequences

Primers	Sequences	Miscellaneous
TERT Ex1 Forward	5'-CGGCCACCCCCGCGATG-3'	Used for cloning (Figure 1E)
TERT Ex16 Reverse	5'-GGGCGGGTGGCCATCAGT-3'	
SSP+TERT Ex1 Forward	5'-TTT CTG TTG GTG CTG ATA TTG C *CGGCCACCCCCGCGATG*-3'	TERT Sequence italicized
VNP+TERT Ex16 Reverse	5'-ACT TGC CTG TCG CTC TAT CTT CT *GGGCGGGTGGCCATCAGT*-3'	
TERT Ex3 Forward	5'- CCGGAAGAGTGTCTGGAGCAAGTTGCAAAGC-3'	
TERT Ex5 Reverse	5'- ACCCTCGAGGTGAGACGCTCGGC-3'	
TERT Ex1 Forward_2	5'- CAGTGCCTGGTGTGCGTG-3'	Paired with either TERT Ex5 Reverse or TERT Ex7 Reverse
TERT Ex5 Reverse	5'- ACGCTGAACAGTGCCTTCAC-3'	Used for total delta 2-4
TERT Ex7 Reverse	5'- GCTGGAGGTCTGTCAAGGTAGAG -3'	Used for Delta 2-4 (with exons 7/8)
MALAT1 Forward	5'- GAATTGCGTCATTTAAAGCCTAGTT-3'	RT-PCR 12 cycles
MALAT1 Reverse	5'- GTTTCATCCTACCACTCCCAATTAAT-3'	
GAPDH Forward	5'-AGCCACATCGCTCAGACAC-3'	RT-PCR 18 cycles
GAPDH Reverse	5'-GCCCAATACGACCAAATCC -3'	
DKK1 Forward	5'-GAGCTACCCGGGTCTTTGTC	RNA-Seq. Validation
DKK1 Reverse	5'-GGGCAGGTTCTTGATAGCGT	
**Probe**	**Sequence**	**Miscellaneous**
TERT Ex1/5 junction probe	5'-6-FAM/TTCCGCCAGGCCGAGCGTCT/3BHQ_1/-3'	

Table S2. Delta 2-4 ddPCR cycles and condition

Temperature (°C)	Time (min)	Cycles
95	10	
94	1	45
64.1	2	
98	5	
12	Hold	
Primer concentration: 900 nM<br>Probe concentration: 250 nM		

Table S3. siRNA Sequences

siRNAs	Sequences	Miscellaneous
siD2-4 (12:12)	5'- UCCUUCCGCCAGGCCGAGCGUCUC-3'	
siD2-4 (8:16)	5'- UCCGCCAGGCCGAGCGUCUCACCU-3'	Chosen siRNA
siD2-4 (16:8)	5'- CCCCUCCUUCCGCCAGGCCGAGCG-3'	

Supplementary Document – Detailed method

MinION TERT Sequencing Library Preparation and Sequencing

Total RNA was extracted (Qiazol) from four human cell lines (iPSCs CHiPs 22 Takara, H1299, Calu-6, A549). Triplicate first strand cDNAs were synthesized with 2 μg of RNA input with oligo dT priming to capture potential protein coding transcripts using SuperScript IV First-Strand Synthesis System (Thermo Fisher). Following cDNA synthesis, cDNAs were amplified with a forward primer that contained the ONT library preparation sequence SSP plus TERT Exon 1 (5'-TTT CTG TTG GTG CTG ATA TTG C *CGGCCACCCCCGCGATG*, (TERT sequence is italicized)) and a reverse primer that contained the ONT library preparation sequence VNP plus TERT Exon 16 (5'-ACT TGC CTG TCG CTC TAT CTT CT *GGGCGGGTGGCCATCAGT*, (TERT sequence is italicized)) for a total 30 cycles. Following confirmation of successful amplification via agarose gel, bands in the 2.5 kb – 4 kb range (upper bands; upper TERT) and bands below 2.5 kb to 0.5 kb (lower bands; lower TERT) were excised and purified via gel extraction (Qiagen). Next, size binned fractions were amplified with ONT barcoded primers 1-12 (4 cells lines by 3 replicates by 2 size bins) for 20 cycles and then excess primers were removed via Ampure XP beads. Following clean up, library preparation was completed by rapid adaptor addition and quantification (agarose gel and qubit DNA) to ensure equal loading. Barcoded libraries from each cell line were pooled at equal molar ratios for each size bin (upper bands or lower bands). The pooled libraries for each size bin were sequenced on its own flow cell (2 R9.4.1 flowcells). The Mk1C MinION device was used for data acquisition. Sequencing was performed with the standard 72 h sequencing protocol run on the MinION Mk1C, using the MinKNOW

software (v 3.3.2). From lower TERT, 21.8 million reads were obtained and 16.4 million reads passed from all 12 samples. From upper TERT, 14.1 million reads were obtained and 12.2 million reads were passed from all 12 samples. Base-calling was performed by Guppy (v 3.1.5). Fastq files which were classified as passed (Phred score of 8) by the MinKNOW software were subsequently processed and analyzed.

Bioinformatics Analyses of TERT Sequencing Library

Passed fastq files were aligned to human genome (GRCh38.p13) using minimap2 (v 2.14; [1, 2]) in SAM MD-tag aware mode. Sam files were sorted with SAMtools (v 1.13; [3]) followed by transcriptClean (v 2.0.2; [4]) for correction of read microindels (< 5 bp), mismatches, and noncanonical splice junctions (< 5 bp). Read annotation was performed with TALON (v 5.0; [5]), using the human Gencode v.38 reference annotation gtf with minimum alignment identity = 0.5 and coverage = 0.5. Identified transcripts were subsequently filtered using a minimum count threshold of N = 35 reads in K = 2 samples. Based on the quantification, Swan (v 2.0; [6]) was used to further process the data. First, a Swan report was generated to screen transcript models. Second, all transcripts were manually binned based on the appearance of their transcript model (visual inspection of inclusion or exclusion of introns or exons at base resolution using IGV). Third, sequences from all transcripts were obtained and confirmed that transcripts were binned correctly by a blinded member of the research team. Based on the binned transcripts, count number (from TALON abundance) of transcripts in the same bin were aggregated and a heatmap was generated using log2-

transformed values. Analyses of the data were performed using python (v 3.9) and R studio (v 2022.07.01; [7]) with the reshape2 package (v1.4.4;[8]), dplyr package (v 1.0.6; [9]), and ggplot2 package (v 3.3.6; [10]).

Bioinformatics Analyses of Delta 2-4 knockdown

Calu-6 cells were treated with scramble non-targeting siRNA controls or an siRNA targeting Delta 2-4 TERT for 48 hours. RNAs were extracted and quality determined by tapestation analysis. Barcoded libraries were prepared following ONT PCR-cDNA kit (SQK-PCB111.24), pooled libraries were prepared by equal molar loading and loaded onto ONT flow cell (FLO-MIN106D) and sequenced for 72 hrs on a MinION Mk1c. Realtime basecalling was performed with Guppy and fastq files were generated as above. Fastq files were aligned to the human genome (GRCh38.p14) and cleaned as described above using minimap2 (v 2.14; [1, 2]), SAMtools (v 1.13; [3]), and transcriptClean (v 2.0.3; [4]). Read annotation was performed with TALON (v 5.0; [5]), using the human Gencode v.44 reference annotation gtf with minimum alignment identity = 0.6 and coverage = 0.8. Identified transcripts were subsequently filtered using a minimum count threshold of N = 5 reads in K = 3 samples. The count matrix generated by TALON (TALON abundance) was filtered. First, ISM (incomplete splice match) and antisense transcripts were removed. Second, transcripts were aggregated by gene names. Third, a custom filter was applied to remove genes that have 0 read counts from any of the three replicates. This filtered count matrix was loaded into DESeq2 for the further analysis (v.1.40.2; [11]). To generate a sample distance heatmap (Euclidean distance), pcaExplorer (v.2.26.1; [12, 13]) was used. Following differential analysis,

differentially expressed genes (DEGs) were identified and vsd transformed values of the genes were used to generate a gene expression heatmap using pheatmap (v.1.0.12; [14]) and viridis (v.0.6.5; [15]). To generate a volcanoplot, p-values and log-fold change (LFC) values from differential analysis was used with EnhancedVolcano (v.1.18.0; [16]), with cut-off being LFC > 1.5 and pvalue < 0.01. Gene set enrichment analysis was carried out with DAVID ([17, 18]) using a list of differentially expressed genes. A list of filtered genes (described above) was used as background genes to reduce the risk of false discovery. Analyses of the data were performed using R studio (v 2023.06.2; [7]).

Cell Culture and Cell Lines

Cellartis® Human iPSC Lines from Takara (ChiPSC22, Cat. No. Y00320) were cultured with strict adherence to manufacturer's protocols and manuals. Cellartis® DEF-CS 500 (Y30017) culture system was employed to maintain iPSC cultures (thawing, passages, media changes and cryopreservation). NSCLC cell lines (A549 (RRID:CVCL_0023), Calu6 (RRID:CVCL_0236), NCI-H1299 (RRID:CVCL_0060), and NCI-H920 (RRID:CVCL_1599)) and Human Bone Osteosarcoma Epithelial Cells (U-2 OS (RRID:CVCL_0042)) were maintained in culture at 37°C in 5% CO_2 in 4:1 DMEM:Medium 199 supplemented with 10% cosmic calf serum (HyClone, Logan, UT). All unmodified cell lines were obtained from American Type Culture Collection (ATCC, Manassas, VA). Human bronchial epithelial cells (HBEC, primary ATCC - PCS-300-010) were maintained in bronchial epithelial growth media (ATCC - PCS-300-030) supplemented with a bronchial epithelial cell growth kit (ATCC - PCS-300-040) on collagen coated plates (porcine gelatin, Millipore Sigma). Cell line identity was verified

by the vendor (ATCC). All cell lines were confirmed to be myocoplasma free at the start of the culture and several clean vials were frozen back for subsequent use (e-Myco kit, Bulldog-Bio). Cells were continuously cultured for 70 passages or up to three months, whichever occurred first, at which point a new mycoplasma free vial was obtained, thawed and cultured.

Plasmid

A codon-optimized Delta 2-4 construct was generated (GeneArt Gene Synthesis, Invitrogen) based on the TERT mRNA sequence (NM_198253.3). The Delta 2-4 sequence was initially inserted into pDONR221, then cloned into pLenti6.2-3xFLAG-V5-ccdB plasmid by Gateway cloning LR recombination reaction (Cat no. 11791019, Invitrogen™) to produce 3xFLAG-V5-Delta 2-4 plasmid. pLenti6.2-3xFLAG-V5-ccdB was a gift from Susan Lindquist & Mikko Taipale (Addgene plasmid # 87072 ; http://n2t.net/addgene:87072 ; RRID:Addgene_87072; [19]).

Stable Cell Line Generation

Stable cell lines expressing Flag-V5-Delta 2-4 were generated for Calu-6 and U-2 OS cells. Lentivirus was produced by transfecting 293T cells (RRID:CVCL_0063) with either pLenti6.2-3xFLAG-V5-ccdB (Control empty vector) or 3xFLAG-V5-Delta 2-4 plasmid, and helper plasmids (psPAX, pMD). Viral supernatants were collected, and target cells (Calu-6 and U-2 OS) were infected. Following infection cells were selected

with Blasticidin (Cat no. R210-01, Gibco) and populations of stably selected cells were cultured and analyzed in subsequent experiments.

Transient siRNA experiments

Calu-6 cells were plated in 6-well plates (300 000 cells per well) and were transfected with non-silencing controls (Santa Cruz Biotechnology, sc-37007) or siRNAs targeting TERT exons 1/5 junction (IDT Integrated DNA Technologies, siD2-4 (12:12): 5'-UCCUUCCGCCAGGCCGAGCGUCUC-3', siD2-4 (8:16): 5'-UCCGCCAGGCCGAGCGUCUCACCU-3', siD2-4 (16:8): 5'-CCCCUCCUUCCGCCAGGCCGAGCG-3'). Calu-6 cells were to transfected with 10 nM of siRNA using Opti-MEM (Gibco) and RNAi max (Invitrogen) for either 48-or-72 hours. Following transfections, cells were washed, trypsinized, counted, and pelleted for downstream analysis.

Reverse transcription-ddPCR

RNA was extracted from frozen cell pellets using RNeasy® Plus Universal Mini Kit (Qiagen, 73404) according to manufacturer's protocol. 1 µg of RNA was used to synthesize cDNAs with the SuperScript IV First-Strand Synthesis System (Thermo Fisher). All cDNAs were diluted 1:4 (20 µL of cDNA + 60 µL nuclease-free water) before use. The cDNAs were used within 48 hours of production in ddPCR measures and stored at -20 °C thereafter. Primer sequences to target *TERT* splice variants (potential FL and minus beta) and methods for calculating percent spliced *TERT* transcripts are from Ludlow et al. [20]. Primers to measure intron 11 retention and intron 14 retention of

TERT are from Dumbović et al. [21]. Primers to measure TERT transcripts containing exons 3 to 5 were Forward: 5'- CCGGAAGAGTGTCTGGAGCAAGTTGCAAAGC-3' targeting exon 3 and Reverse: 5'- ACCCTCGAGGTGAGACGCTCGGC-3' targeting exon 5. For intron 11 retention, intron 14 retention, and TERT exons 3 to 5, EvaGreen Supermix (Cat no. 186-4034, BIORAD) was used with a two-step PCR where the annealing/elongation temperature was 60 °C for a minute.

For the delta 2-4 assays, primers to target TERT total delta 2-4 (Delta 2-4 and delta 2-4/ delta 7-8) are Forward: 5'- CAGTGCCTGGTGTGCGTG-3' targeting exon 1 and Reverse: 5'- ACGCTGAACAGTGCCTTCAC-3' targeting exon 5. Primers to target Delta 2-4 are Forward: 5'- CAGTGCCTGGTGTGCGTG -3' targeting exon 1 (same as total delta 2-4) and Reverse: 5'- GCTGGAGGTCTGTCAAGGTAGAG -3' targeting exon 7. To quantify skipping of *TERT* exons 2-4, a 1/5 junction 5' hydrolysis probe was used: 5'-6-FAM/TTCCGCCAGGCCGAGCGTCT/3BHQ_1/-3'. For the delta 2-4 assays, primer concentration was 900 nM and probe concentration was 250 nM. Optimized PCR thermal cycling specifically for delta 2-4 assays was: an initial incubation at 95°C for 10 min, 45 cycles of 1 min at 94°C and 2 min at 64.1°C, followed by a final incubation at 98°C for 5 min and held at 12°C. The amplification signals were read using the QX200™ Droplet Reader and analyzed using its associated QuantaSoft software (Bio-Rad).

Cloning method to identify TERT mRNA variants

Using oligo dT primed cDNAs from A549 cells (total RNA input 2 µg, Superscript IV), PCR was performed with TERT exon 1 (5'-CGGCCACCCCCGCGATG-3') and exon 16

(5’-GGGCGGGTGGCCATCAGT-3’) targeting primers with a high-yield and high-fidelity polymerase (Advantage® GC 2 PCR Kit, Cat. No. 639120, TaKaRa). The PCR products were TA cloned into TOPO™ TA and transformation was performed using TOP 10 E.coli (Termo Fisher). EcoRI digests identified clones that had inserts shorter than FL TERT. Identified unique plasmids were Sanger sequenced with M13 F and M13 R to verify sequences and aligned to TERT using freely available sequence analysis tools. Delta 2-4 and Delta 2-4/delta 7-8 TERT were identified using these methods.

Nuclear and Cytoplasm fragmentation

Calu-6 cells (n = 3) were used to obtain cytoplasmic RNA and nuclear RNA fractions using PARIS™ Kit (Cat no. AM1921, Invitrogen™). Following RNA extraction, TURBO DNA-free™ Kit (Cat no. AM1907, Invitrogen™) was used to remove trace DNA contamination. Cytoplasmic and nuclear localization of MALAT1 and GAPDH were measured by gel-based PCR using 2 µL of cDNA (equivalent to 25 ng of RNA input) and 2x EmeraldAmp® MAX HS PCR Master Mix (Cat no. RR330, TaKaRa). Primers to target MALAT1 were Forward: 5’- GAATTGCGTCATTTAAAGCCTAGTT-3’ and Reverse: 5’- GTTTCATCCTACCACTCCCAATTAAT-3’. Primers to target GAPDH were Forward: 5’-AGCCACATCGCTCAGACAC-3’ and Reverse: 5’-GCCCAATACGACCAAATCC -3’. Annealing temperature was 60 °C for 30 seconds for both targets, and cycle numbers were 12 for MALAT1 and 18 for GAPDH. TERT primers and PCR conditions were described above.

Droplet Digital TRAP Assay for Telomerase Activity

Quantification of telomerase enzyme activity was determined by the droplet digital TRAP assay [22, 23]. In brief, a 1.0 x 10^6 cell pellet was lysed in 40 μL of NP-40 lysis buffer, diluted to a final concentration of 1.25 x 10^3 cells/μL, and 2 μL added to an extension reaction (50 cell equivalents per μL) for 60 min followed by a 5 min heat inactivation of telomerase at 95°C. An aliquot (2 μL) of extension products containing an equivalent of 100 cells was amplified in a droplet digital PCR for 40 cycles. Droplet fluorescence intensity and number were read and counted on the QX200 Droplet Reader (Bio-Rad). Data were calculated to represent telomerase extension products per cell equivalent.

Terminal Restriction Fragment Assay for Telomere Length Analysis

The average length of telomeres (terminal restriction fragment lengths) was measured as previously described [24] with the following modifications: A DIG-labeled DNA molecular weight marker II ladder was loaded on either side of the samples (Millipore Sigma, St. Louis, MO). DNA was transferred to Hybond-N+ membranes (GE Healthcare, Piscataway, NJ) using overnight transfers. The membrane was briefly air-dried and DNA was fixed by UV-crosslinking. Membranes were then probed for telomeres using a digoxigenin (DIG)-labeled telomere probe, detected with a horseradish peroxidase-linked anti-DIG antibody (Cat no. 11093274910; Roche, Basel, Switzerland, RRID:AB_2734716), exposed with CDP-star (Cat no. 11759051001; Roche), and imaged (Chemidoc XRS + Molecular Imager, Bio-Rad).

Clonogenic Assay

Calu-6 and U-2 OS cells with or without TERT Delta 2-4 overexpression were used for clonogenic assays. Calu-6 cells were treated with siRNAs (control and Delta 2-4 targeting) in duplicate (10 nM siRNA). After 24 hours of siRNA and treatments, cells were trypsinized, counted, and plated in triplicate at densities of 100 cells per well in a 6-well plate resulting in 6 replicates for each condition. Media was changed every four days, and once clearly visible colonies were present (~11 days), they were fixed and stained with Crystal violet [20] . Plates were imaged (Chemidoc XRS + Molecular Imager, Bio-Rad) and the number of colonies were counted by ColonyCountJ [25].

Alamar Blue cell viability Assay

To determine the viability of cells with overexpression of Delta 2-4, RNAi and cisplatin treatment, we used Alamar blue assays. In TERT Delta 2-4 expressing U-2 OS and Calu-6 cells, 1.0 x 10^4 cells were plated on 96-well plates in 180 µL of growth media. Immediately after plating the cells, 20 µL of either PBS (vehicle) or cisplatin (Cat. No. 232120-50MG, Millipore Sigma) dissolved in PBS was added to wells at 6 different concentrations (U-2 OS: 6.25-4.69-3.52-2.64-1.98-0.39 µg/mL; Calu-6: 100-25-6.25-1.56-0.39-0.1 µg/mL). After 44 hours of treatment exposure, 20 µL of 10X Alamar blue was added to the media. After 4 hours of incubation, the fluorescence of each well was determined on a plate reader (Molecular Devices SpectraMax iD3) at 545 nM excitation and 590 nM emission. Values from cisplatin treated cells were divided by averaged

values from PBS treated cells from the same cell line to calculate percentage of alive cells (with the vehicle condition assumed to be 100% viable).

For siRNA/rescue experiment in Calu-6, reverse transfection was performed [26]. Transfection complexes were prepared with siRNAs (siD2-4 (8:16)) using Opti-MEM (Gibco) and RNAi max (Invitrogen) following the manufacturer's procedures and 20 µL of the complex was plated in 96-well plate and incubated for 20 minutes. 1.0 x 10^4 cells were plated on each well in 160 µL of growth media, followed by 20 µL of either PBS or cisplatin (1.59 µg/mL) dissolved in PBS. Final concentration of siRNA was 30 nM in 200 µL. After 44 hours, 20 µL of 10X Alamar blue (Cat. No. DAL1025, Invitrogen™) was added. After 4 hours of incubation, the fluorescence of each well was determined on a plate reader (Molecular Devices SpectraMax iD3) at 545 nM excitation and 590 nM emission. Viability was calculated as describe above.

Western Blot Analysis

Cell pellets (1 x 10^6 cells) were collected and lysed in 100 µL of lysis buffer (NP40 buffer – 10 mM Tris-HCl pH 8.0, 1mM $MgCl_2$, 1mM EDTA, 1% NP-40, 0.25 mM Sodium deoxycholate, 10% Glycerol, 150 mM NaCl, 0.036% 2-mercapto ethanol, 0.2 mM). Total protein lysates were further treated with 100 µL of 2 x Laemmli buffer (Bio-Rad) and boiled for 10 mins at 95°C. Each protein lysate was loaded by equal volume and resolved by SDS-polyacrylamide gel electrophoresis, transferred to polyvinylidene fluoride (PVDF) membranes, and detected with antibodies for TERT (rabbit monoclonal,

Y182, Cat. No. ab32020, Abcam, 1:1 000 dilution in 5% BSA), FLAG (rabbit monoclonal, D6W5B, Cat. No. 14973, Cell Signaling, 1:1 000 dilution in 5% BSA), and V5 (mouse monoclonal, SV5-Pk1, Cat. No. R960-25, 1:1 000 dilution in 5% BSA). Protein loading was determined with antibodies against beta-actin (mouse monoclonal [8H10D10], 3700, Cell Signaling Technology, 1:1 000 in 5% BSA). Blots were imaged with Bio-Rad ChemiDoc XRS+ Molecular Imager and quantified with Bio-Rad Image Lab software.

Growth Curve – Population Doubling (PD)

To track and compare growth rates of stable cells lines of control vector and TERT Delta 2-4 overexpressing cells, population doublings were calculated based on the formula below. PD values were started from 10 doublings, after 2 weeks of blasticidin selection and PD values were calculated accumulatively per subculture based on the formula: new PD = (LOG([the number of trypsinized cells]/[the number of previously plated cells])/0.3)+ [the number of previous PD].

Immunostaining

For immunocytochemistry, cells were plated on Nunc™ Lab-Tek™ II Chamber Slide™ (Thermo fisher scientific, Cat# 154526) and were fixed with 4% PFA for 10 min and washed three times with cold PBS. After incubation of PBST (0.5% Triton X-100 in PBS) for 15 min, the cells were blocked with blocking solution (3% bovine serum albumin, and 0.1% Tween 20 in PBS) for 30 min followed by incubation with primary antibodies (FLAG: monoclonal rabbit, Cell signaling, Cat# 14793, 1:400 dilution; COX4:

monoclonal mouse, cell signaling, Cat# 11967, 1:200 dilution) diluted in blocking solution for 1 h. Cells were washed three times with PBST and incubated with secondary antibodies (Anti-rabbit IgG Alexa Flour 488 Conjugate #4412, Anti-mouse IgG Alexa Flour 594 Conjugate #8889, Cell signaling) in blocking solution for 30 min, washed twice with PBST. The cover glass was mounted on a slide glass with ProLong Gold Antifade Mountant with DNA stain DAPI (Invitrogen, Cat# P36941). Images were acquired from a confocal microscope at 40x magnification (Lecia, Stelaris).

Statistical analysis

For *TERT* transcript subcellular localization experiment (Fig. 1G) and siRNA testing experiments (Fig. S2B-D), one-way ANOVA with uncorrected Fisher's LSD for post hoc comparisons were used to determine statistical significance between experimental groups. For knockdown and rescue experiments (Fig. 3D,E and Fig. S3D, E), p-value was calculated using one-way ANOVA with Tukey's multiple comparison test compared to every other condition. When a *p*-value from a comparison between two groups was $p < 0.05$, the two groups were assigned different letters of the alphabet (i.e., a vs. b). When the *p*-value was $p \geq 0.05$, two groups were assigned to the same letter (a vs. a). To compare two conditions, unpaired two-tailed t-test was used to calculate *p*-values.

1 Li H. New strategies to improve minimap2 alignment accuracy. *Bioinformatics* 2021; 37: 4572-4574.

2 Li H. Minimap2: pairwise alignment for nucleotide sequences. *Bioinformatics* 2018; 34: 3094-3100.

3 Danecek P, Bonfield JK, Liddle J, Marshall J, Ohan V, Pollard MO *et al*. Twelve years of SAMtools and BCFtools. *GigaScience* 2021; 10.

4 Wyman D, Mortazavi A. TranscriptClean: variant-aware correction of indels, mismatches and splice junctions in long-read transcripts. *Bioinformatics* 2018; 35: 340-342.

5 Wyman D, Balderrama-Gutierrez G, Reese F, Jiang S, Rahmanian S, Forner S *et al*. A technology-agnostic long-read analysis pipeline for transcriptome discovery and quantification. *bioRxiv* 2020: 672931.

6 Reese F, Mortazavi A. Swan: a library for the analysis and visualization of long-read transcriptomes. *Bioinformatics* 2021; 37: 1322-1323.

7 RStudio Team. RStudio: Integrated Development Environment for R. RStudio, PBC, 2020.

8 Wickham H. Reshaping Data with the reshape Package. *Journal of Statistical Software* 2007; 21: 1--20.

9 Wickham H, François R, Henry L, Müller K, Vaughan D. dplyr: A Grammar of Data Manipulation, 2023.

10 Wickham H. *ggplot2: Elegant Graphics for Data Analysis*. Springer-Verlag New York, 2016.

11 Love MI, Huber W, Anders S. Moderated estimation of fold change and dispersion for RNA-seq data with DESeq2. *Genome Biol* 2014; 15: 550.

12 Marini F, Binder H. pcaExplorer: an R/Bioconductor package for interacting with RNA-seq principal components. *BMC Bioinformatics* 2019; 20: 331.

13 Ludt A, Ustjanzew A, Binder H, Strauch K, Marini F. Interactive and Reproducible Workflows for Exploring and Modeling RNA-seq Data with pcaExplorer, Ideal, and GeneTonic. *Curr Protoc* 2022; 2: e411.

14 Kolde R. pheatmap: Pretty Heatmaps, 2019.

15 Pedro A. viridis(Lite) - Colorblind-Friendly Color Maps for R, 2024.

16 Blighe K, Rana S, Lewis M. EnhancedVolcano: Publication-ready volcano plots with enhanced colouring and labeling, 2023.

17 Huang da W, Sherman BT, Lempicki RA. Systematic and integrative analysis of large gene lists using DAVID bioinformatics resources. *Nat Protoc* 2009; 4: 44-57.

18 Huang da W, Sherman BT, Lempicki RA. Bioinformatics enrichment tools: paths toward the comprehensive functional analysis of large gene lists. *Nucleic Acids Res* 2009; 37: 1-13.

19 Taipale M, Tucker G, Peng J, Krykbaeva I, Lin ZY, Larsen B *et al*. A quantitative chaperone interaction network reveals the architecture of cellular protein homeostasis pathways. *Cell* 2014; 158: 434-448.

20 Ludlow AT, Wong MS, Robin JD, Batten K, Yuan L, Lai TP *et al*. NOVA1 regulates hTERT splicing and cell growth in non-small cell lung cancer. *Nat Commun* 2018; 9: 3112.

21 Dumbovic G, Braunschweig U, Langner HK, Smallegan M, Biayna J, Hass EP *et al*. Nuclear compartmentalization of TERT mRNA and TUG1 lncRNA is driven by intron retention. *Nat Commun* 2021; 12: 3308.

22 Ludlow AT, Robin JD, Sayed M, Litterst CM, Shelton DN, Shay JW *et al*. Quantitative telomerase enzyme activity determination using droplet digital PCR with single cell resolution. *Nucleic Acids Res* 2014; 42: e104.

23 Sayed ME, Slusher AL, Ludlow AT. Droplet Digital TRAP (ddTRAP): Adaptation of the Telomere Repeat Amplification Protocol to Droplet Digital Polymerase Chain Reaction. *Journal of visualized experiments : JoVE* 2019.

24 Mender I, Shay JW. Telomere Restriction Fragment (TRF) Analysis. *Bio Protoc* 2015; 5.

25 Maurya DK. ColonyCountJ: A User-Friendly Image J Add-on Program for Quantification of Different Colony Parameters in Clonogenic Assay. *Journal of Clinical Toxicology* 2017; 7: 2161-0495.1000358.

26 Ovcharenko D, Jarvis R, Hunicke-Smith S, Kelnar K, Brown D. High-throughput RNAi screening in vitro: from cell lines to primary cells. *RNA* 2005; 11: 985-993.
