# Supplementary material for: Discovery and characterization of a novel telomerase alternative splicing isoform that protects lung cancer cells from chemotherapy induced cell death
Source: Sci Rep. 2025 Feb 25;15:6787. doi: 10.1038/s41598-025-90639-3 (PMC11861669; doi:10.1038/s41598-025-90639-3)
Supplement: Supplementary file 2 — Supplementary Information 2. [file 41598_2025_90639_MOESM2_ESM.pptx]

## Slide 1
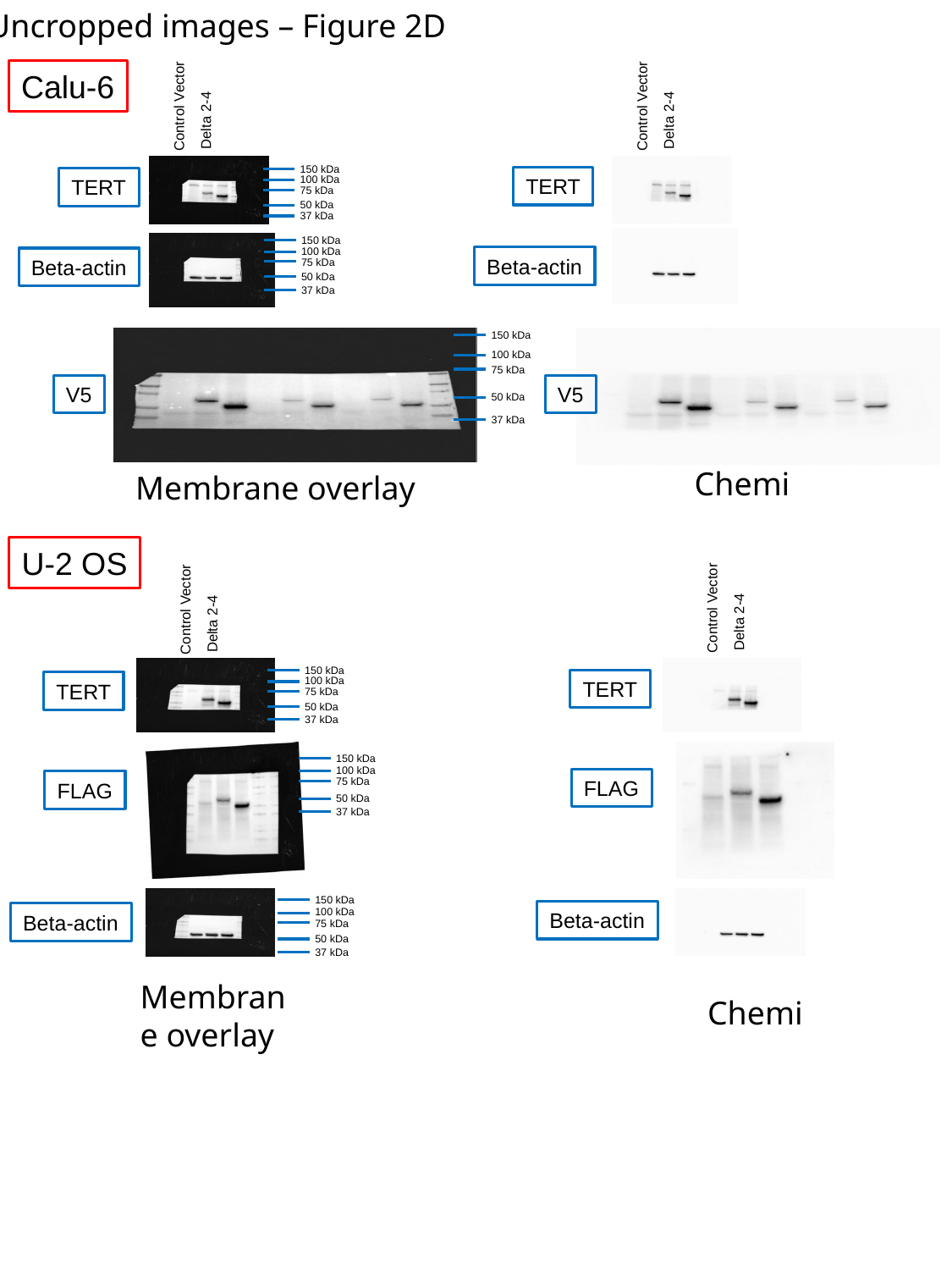

Uncropped images – Figure 2D
Calu-6
Delta 2-4
Delta 2-4
Control Vector
Control Vector
150 kDa
100 kDa
TERT
TERT
75 kDa
50 kDa
37 kDa
150 kDa
100 kDa
Beta-actin
Beta-actin
75 kDa
50 kDa
37 kDa
150 kDa
100 kDa
75 kDa
V5
V5
50 kDa
37 kDa
Chemi
Membrane overlay
U-2 OS
Delta 2-4
Delta 2-4
Control Vector
Control Vector
150 kDa
100 kDa
TERT
TERT
75 kDa
50 kDa
37 kDa
150 kDa
100 kDa
75 kDa
FLAG
FLAG
50 kDa
37 kDa
150 kDa
100 kDa
Beta-actin
Beta-actin
75 kDa
50 kDa
37 kDa
Membrane overlay
Chemi

## Slide 2
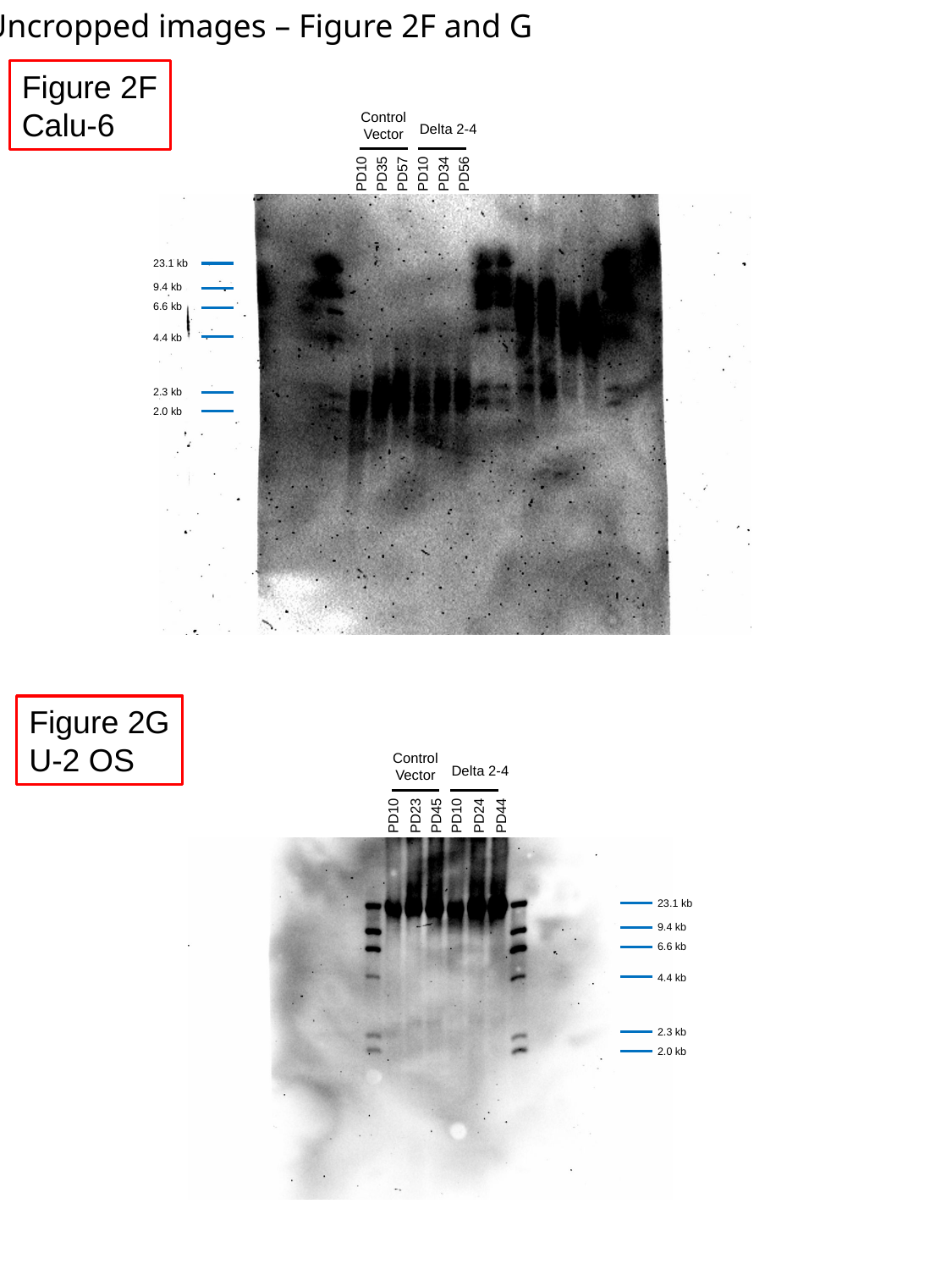

Uncropped images – Figure 2F and G
Figure 2F
Calu-6
Control
Vector
Delta 2-4
PD35
PD57
PD34
PD56
PD10
PD10
23.1 kb
9.4 kb
6.6 kb
4.4 kb
2.3 kb
2.0 kb
Figure 2G
U-2 OS
Control
Vector
Delta 2-4
PD23
PD45
PD24
PD44
PD10
PD10
23.1 kb
9.4 kb
6.6 kb
4.4 kb
2.3 kb
2.0 kb

## Slide 3
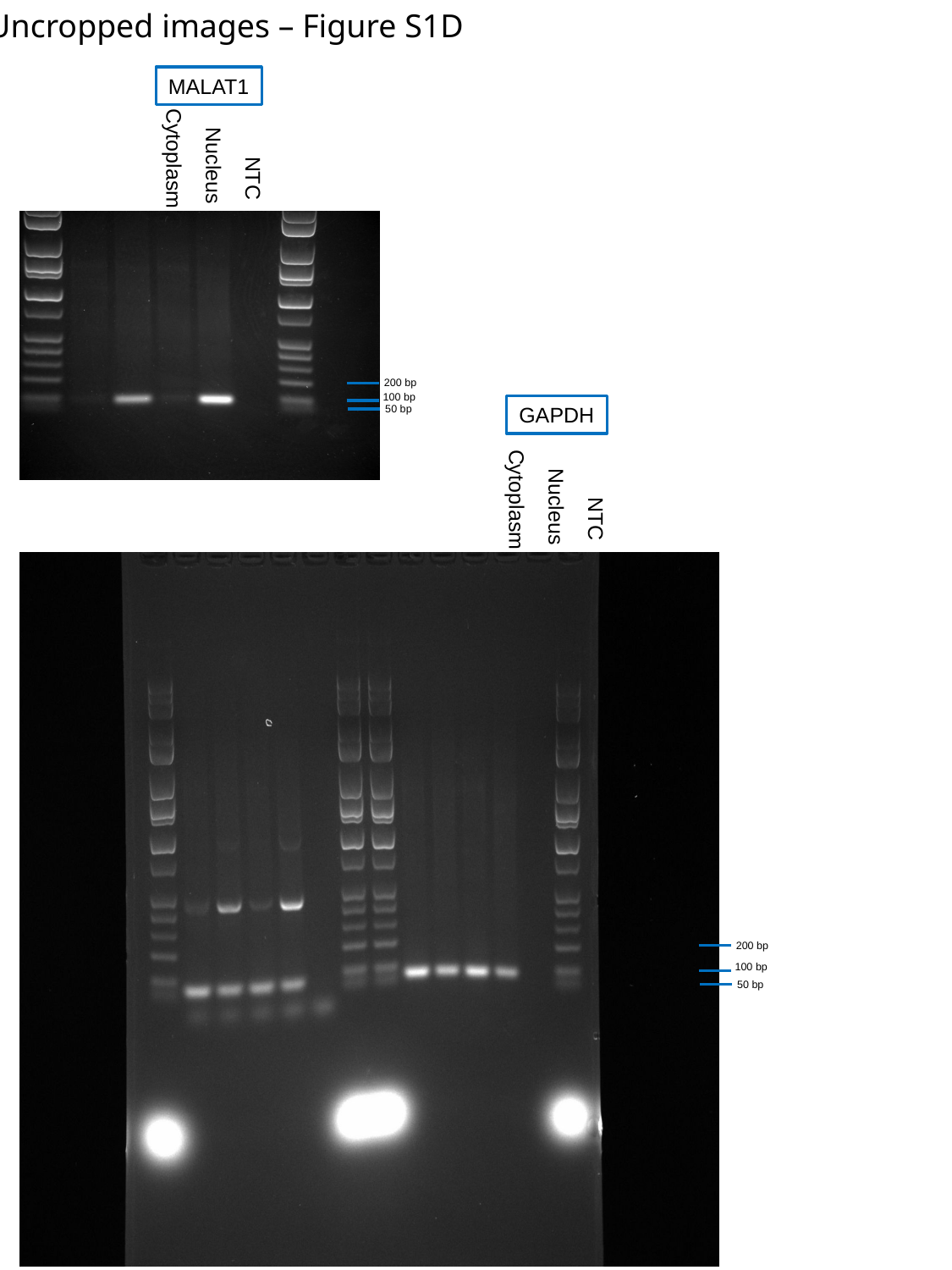

Uncropped images – Figure S1D
MALAT1
Cytoplasm
Nucleus
NTC
200 bp
100 bp
50 bp
GAPDH
Cytoplasm
Nucleus
NTC
200 bp
100 bp
50 bp

## Slide 4
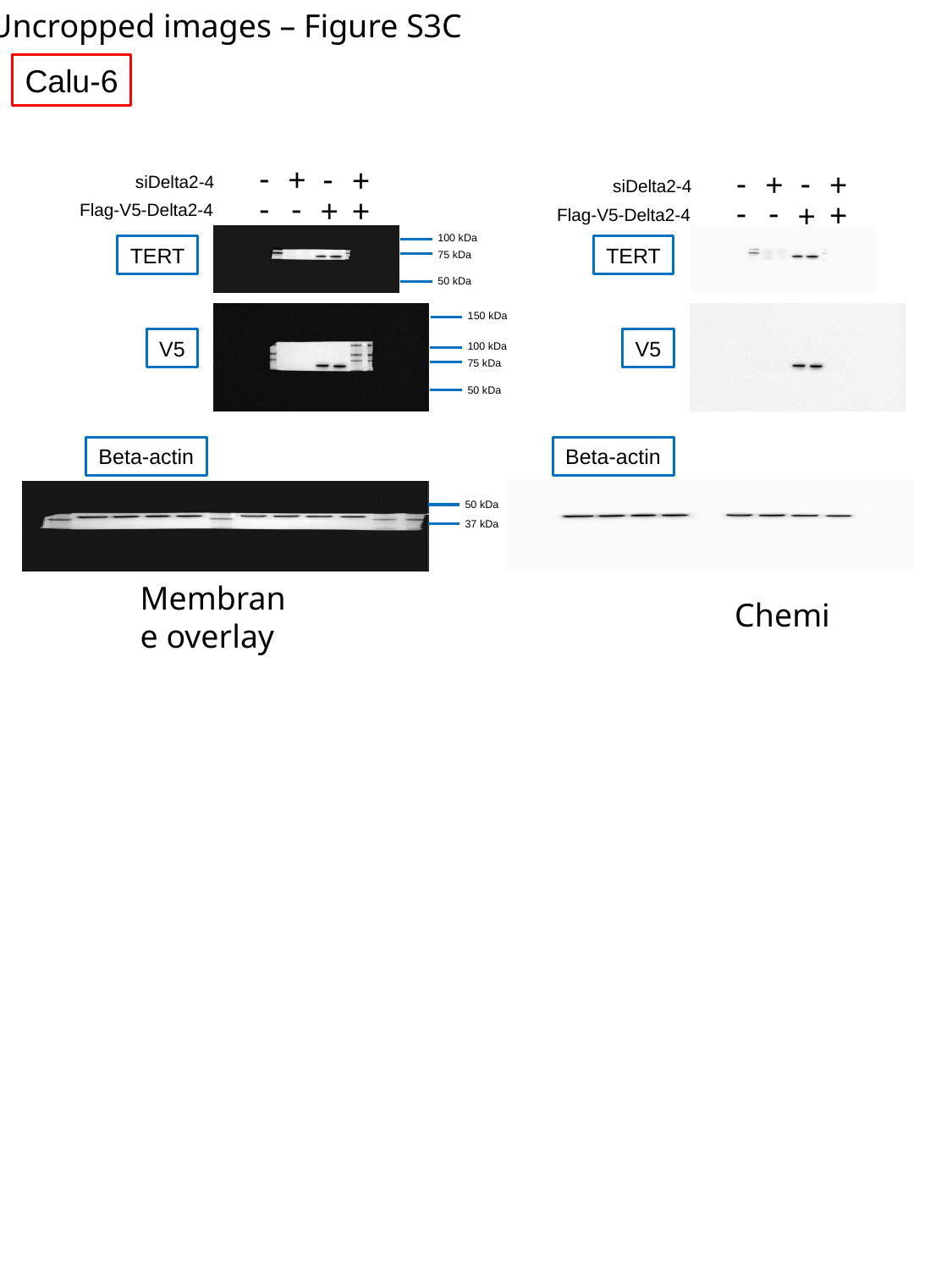

Uncropped images – Figure S3C
Calu-6
-
-
+
+
-
-
+
+
siDelta2-4
siDelta2-4
-
-
+
+
-
-
+
+
Flag-V5-Delta2-4
Flag-V5-Delta2-4
100 kDa
TERT
TERT
75 kDa
50 kDa
150 kDa
V5
V5
100 kDa
75 kDa
50 kDa
Beta-actin
Beta-actin
50 kDa
37 kDa
Membrane overlay
Chemi
